# Temporal dynamics of the fecal microbiota in veal calves in a 6-month field trial

**DOI:** 10.1186/s42523-020-00052-6

**Published:** 2020-09-15

**Authors:** Méril Massot, Marisa Haenni, Thu Thuy Nguyen, Jean-Yves Madec, France Mentré, Erick Denamur

**Affiliations:** 1 Université de Paris, IAME, INSERM, Site Xavier Bichat, 16 rue Henri Huchard, F-75018 Paris, France; 2 Unité Antibiorésistance et Virulence Bactériennes, Université de Lyon - ANSES, Laboratoire de Lyon, Lyon, France; 3 AP-HP, Hôpital Bichat-Claude Bernard, Département d’Epidémiologie, Biostatistiques et Recherche Clinique, F-75018 Paris, France; 4 AP-HP, Hôpital Bichat-Claude Bernard, Laboratoire de Génétique Moléculaire, F-75018 Paris, France

**Keywords:** Veal calves, Fecal microbiota development, *Escherichia coli*, 16S rRNA gene sequencing, Milk powder, Antibiotics

## Abstract

**Background:**

Little is known about maturation of calves’ gut microbiome in veal farms, in which animals are confined under intensive-farming conditions and the administration of collective antibiotic treatment in feed is common. We conducted a field study on 45 calves starting seven days after their arrival in three veal farms. We collected monthly fecal samples over six months and performed 16S rRNA gene sequencing and quantitative PCR of *Escherichia coli* to follow the dynamics of their microbiota, including that of their commensal *E. coli* populations. We used mixed-effect models to characterize the dynamics of α-diversity indices and numbers of *E. coli*, and searched for an effect of collective antibiotic treatments on the estimated parameters. On two farms, we also searched for associations between recommended daily doses of milk powder and bacterial abundance.

**Results:**

There was high heterogeneity between calves’ microbiota upon their arrival at the farms, followed by an increase in similarity, starting at the first month. From the second month, 16 genera were detected at each sampling in all calves, representing 67.5% (± 9.9) of their microbiota. Shannon diversity index showed a two-phase increase, an inflection occurring at the end of the first month. Calves receiving antibiotics had a lower intercept estimate for Shannon index (− 0.17 CI_95%_[-0.27; − -0.06], *p* = 0.003) and a smaller number of *E. coli*/ gram of feces during the treatment and in the 15 days following it (− 0.37 log_10_ (*E. coli*/g) CI_95%_[− 0.66; − 0.08], *p* = 0.01) than unexposed calves. There were moderate to strong positive associations between the dose of milk powder and the relative abundances of the genera *Megasphaera, Enterococcus, Dialister* and *Mitsuokella*, and the number of *E. coli* (r_s_ ≥ 0.40; Bonferroni corrected *p* < 0.05).

**Conclusions:**

This observational study shows early convergence of the developing microbiota between veal calves and associations between the dose of milk powder and members of their microbiota. It suggests that administration of collective antibiotic treatment results in a reduction of microbial diversity and size of the *E. coli* population and highlights the need for additional work to fully understand the impact of antibiotic treatment in the veal industry.

## Background

The veal-calf industry is an intensive farming system that produces meat from milk-fed calves. Europe is one of the largest producer of veal in the world, producing approximately six million heads yearly, mostly in France, the Netherlands, Italy, and Belgium. Male dairy calves are commonly used to produce veal. They are purchased in dairy farms by “integrators”, companies that are involved in all stages of the production process [1]. They are collected at 2 weeks of age, batched, and placed without delay in dedicated closed buildings for approximately 6 months. Calves are mainly fed milk replacers and a small amount of solid feed is introduced during the first weeks for the welfare of the animals. They are reared indoors, often in high-animal-density buildings, which increases the risk of pathogens spreading among the batch. Collective antibiotic treatment is frequent, particularly at the start of the fattening period, when calves coming from different dairy farms are grouped together. In France, veal calves typically receive more than eight antibiotic treatments during the fattening process, mainly by the oral route [2, 3]. Collective treatment represents almost 96% of the administrated treatments and 88% of the prescriptions are made in advance of an anticipated outbreak of disease, when some animals in the batch develop symptoms of infection [2–4]. A pervasive effect of antibiotic treatment can be the collapse of gut bacterial populations, which results in a loss of fecal microbial diversity, as shown in cattle [5, 6].

Because of the extensive use of antibiotics, combined with specific dietary practices and intensive farming practices, maintaining a proper gut microbiota balance during calf development is a key challenge in the veal-calf industry. To our knowledge, only a few attempts have been made to investigate the effect of antibiotics on the gut microbiota composition of pre-weaned dairy calves in commercial farms [7, 8], and no study has focused on calves reared in veal farms.

Based on a field trial, we report the temporal dynamics of bacterial communities in veal calves highly exposed to antibiotics from an early age, with an additional focus on the commensal *E. coli* population. Although *E. coli* is one of the most common pathogens that causes diarrhea in calves, it is also found as a commensal in the gut of healthy calves [9, 10]. Few studies have focused on commensal *E. coli* populations in pre-weaned calves [11]. We hypothesized that the developmental trajectory of the microbiota is influenced by the use of antibiotics during growth, resulting in the development of a community with lower diversity and persistent shifts in taxonomic composition. We addressed this question by performing 16S rRNA gene sequencing and quantitative PCR (qPCR) of *Escherichia* on 312 rectal swabs collected from 45 veal calves distributed in French veal farms over 6 months.

## Results

### Animal sampling and follow-up

**Fig. 1:**
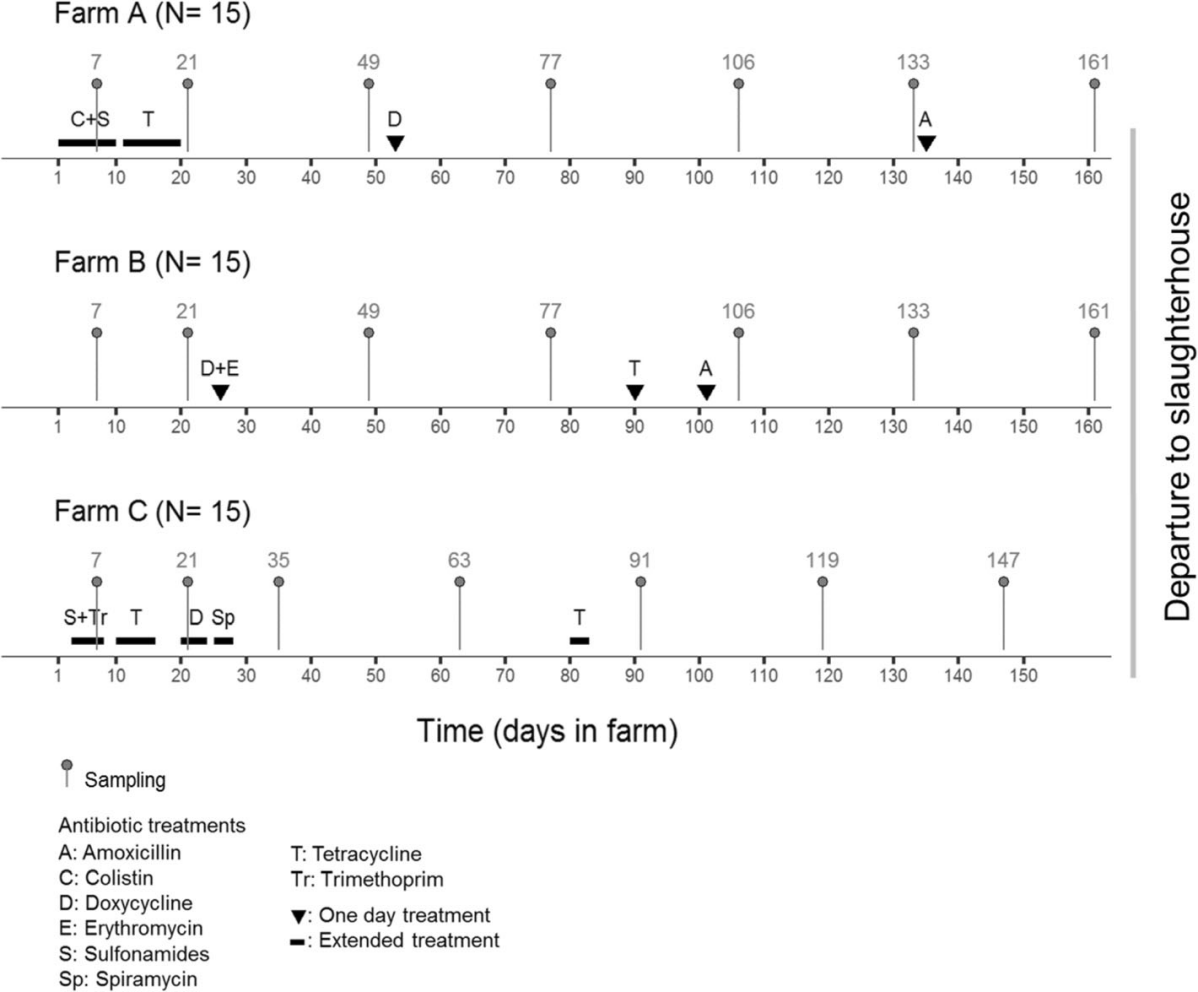
Scheme of sampling dates for each farm. Sampling points for farm A, farm B, and farm C are represented in the upper panel, middle panel, and lower panel, respectively. “N” indicates the number of calves studied on each farm. The days of sampling are indicated by grey dots. Of note, one calf on farm C died during the fattening period and was excluded from the study. Antibiotic treatments are indicated by bold dark lines or back triangles, and the names of the antibiotics are given in the legend

We collected fecal samples from 15 calves each from three veal farms partnered with different integrators in Brittany, France, during fattening. They were sampled by rectal swabbing at days 7 and 21 and then monthly for 5 months until the departure of the batch to the slaughterhouse. The calves were 14 days old when they arrived on the farms and were mainly fed milk replacer throughout the fattening period, which is reconstituted from milk powder with hot water. Calves stayed for 161 days on farms A and B and 147 days on farm C. Collective antibiotic treatments were recorded by the three farms throughout the fattening period (Fig. 1). Antibiotics were always used at therapeutic doses and administered orally in the feed. All calves received antibiotics more than once and calves from farms A and C received several consecutive antibiotic treatments during the first month (Fig. 1).

All calves from farms A and B included in the study were followed until the end of the fattening period. One calf from farm C died during fattening and was excluded from the study. For the 44 remaining calves, there were no missing samples during the follow-up and thus downstream analyses were performed on 308 samples.

After sampling, swabs were placed immediately in portable coolers with ice packs. Swabs were shipped to the antimicrobial resistance and virulence lab of the French Agency for Food, Environmental and Occupational Health & Safety (ANSES) lab in Lyon, France, within 24 h, and stored at − 80 °C. After genomic DNA extraction from the swabs, we characterized the fecal microbiota by 16S rRNA gene amplicon sequencing (V4 region) using Illumina MiSeq technology. *E. coli* was quantified by qPCR targeting the 16S rRNA gene sequence specific to the *Escherichia* genus.

### 16S amplicon sequencing

After processing reads using the mothur pipeline, 34,153,188 quality amplicons were generated, with an average of 111,248 ± 63,002 per sample (Additional file 1: Fig. S1a). One sample had mostly poor-quality reads and a very small number of amplicons and was thus excluded from the downstream 16S amplicon sequencing analyses. The minimum number of operational taxonomic units (OTUs) detected in a sample was 119 and the maximum 1302 (Additional file 1: Fig. S1b). The rarefaction threshold was set to 47,000 sequences (Additional file 1: Fig. S1c). Seven samples were below this threshold and excluded from the α- and β-diversity analyses.

### Weighted and unweighted Unifrac distances

**Fig. 2:**
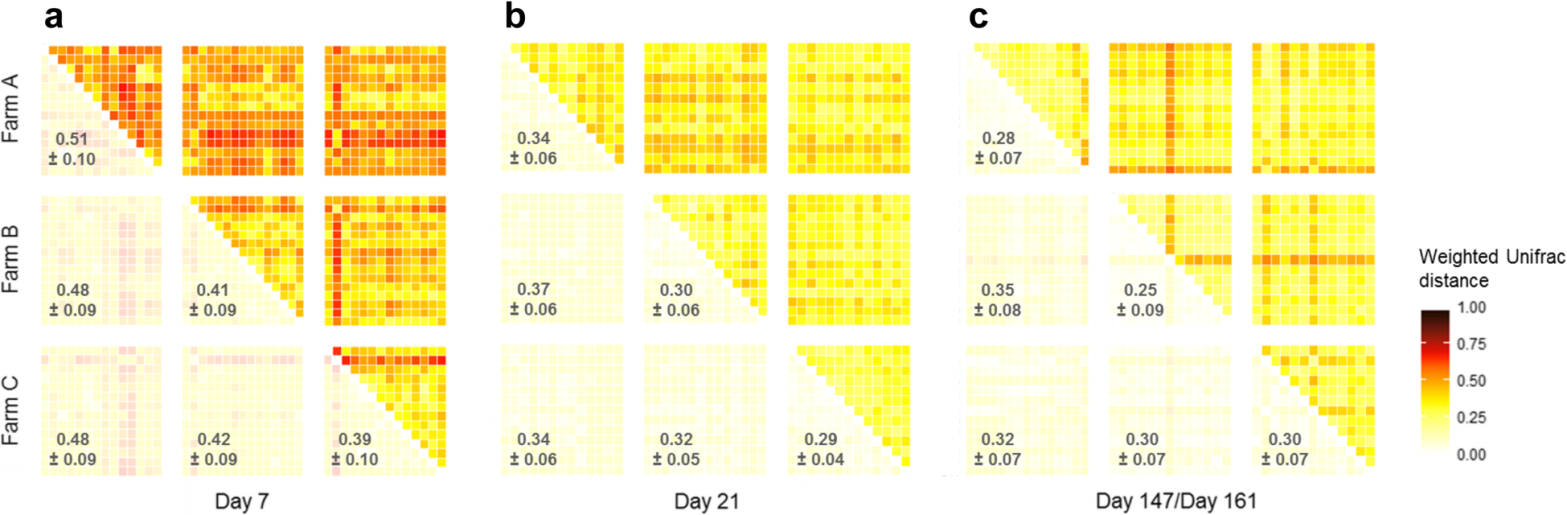
Heatmaps of the weighted Unifrac distances at the first month and last month of fattening. Heatmaps of the β-diversity weighted Unifrac distances matrix are shown for the (**a**) first sampling (day 7), (**b**) second sampling (day 21), and (**c**) last sampling (day 161 for farms A and B and day 147 for farm C). Each square represents a pairwise distance between two calves. The pale yellow squares indicate low Unifrac distances, whereas dark red squares indicate high Unifrac distances. The calves are ordered according to the farms in both the lines and columns. The calf’s distance from itself is represented by the white square on the main diagonal. The means ± standard deviations for each sampling and farm are shown in the lower triangles

The similarity of microbiota composition among calves was tracked using β-diversity measures, which represents the dissimilarity between samples. The weighted Unifrac distances between calves were the highest on day 7, with an overall mean of 0.45 (± 0.10) (Fig. 2a). The weighted Unifrac distances started to decrease at the next sample, the overall mean reaching 0.33 (± 0.06) on day 21 (Fig. 2b). Weighted Unifrac distances remained low until the end of fattening, with a mean of 0.31 (± 0.08) on the last day (Fig. 2c). The time of sampling had a significant effect on the calf microbiome composition (*p* = 0.001, permutational multivariate analysis of variance (PERMANOVA) on weighted Unifrac distances). It explained 15.5% of the between-sample variation in calves, indicating extensive sharing of the microbial community among calves at a given time of the fattening period. The microbiota of individuals belonging to the same farm were no more similar than those of individuals from different farms (*p* = 0.4, PERMANOVA on weighted Unifrac distances). Despite the use of different antibiotic molecules and different times of collective antibiotic treatments between farms, the farm affiliation explained only 4.0% of the between sample variation (Fig. 2).

We obtained similar results for the unweighted Unifrac distances (Additional file 2: Fig. S2, *p* = 0.001 and *p* = 0.5 for the time of sampling and farm in the PERMANOVA test, respectively). The farm affiliation explained 2.9% of the between-sample variation.

The weighted Unifrac distances between consecutive samplings showed a trend over time, with a downward trajectory for almost all calves (Additional file 3: Fig. S3). The mean intra-calf weighted Unifrac distances between the first and second samplings and the second and third samplings was 0.40 (± 0.10) and 0.33 (± 0.08), respectively. The mean intra-calf weighted Unifrac distances between the second to last sampling and last sampling was 0.25 (± 0.07). These results suggest that the magnitude of changes tended to decrease on a monthly scale.

### Taxonomic composition of the microbiota

**Fig. 3:**
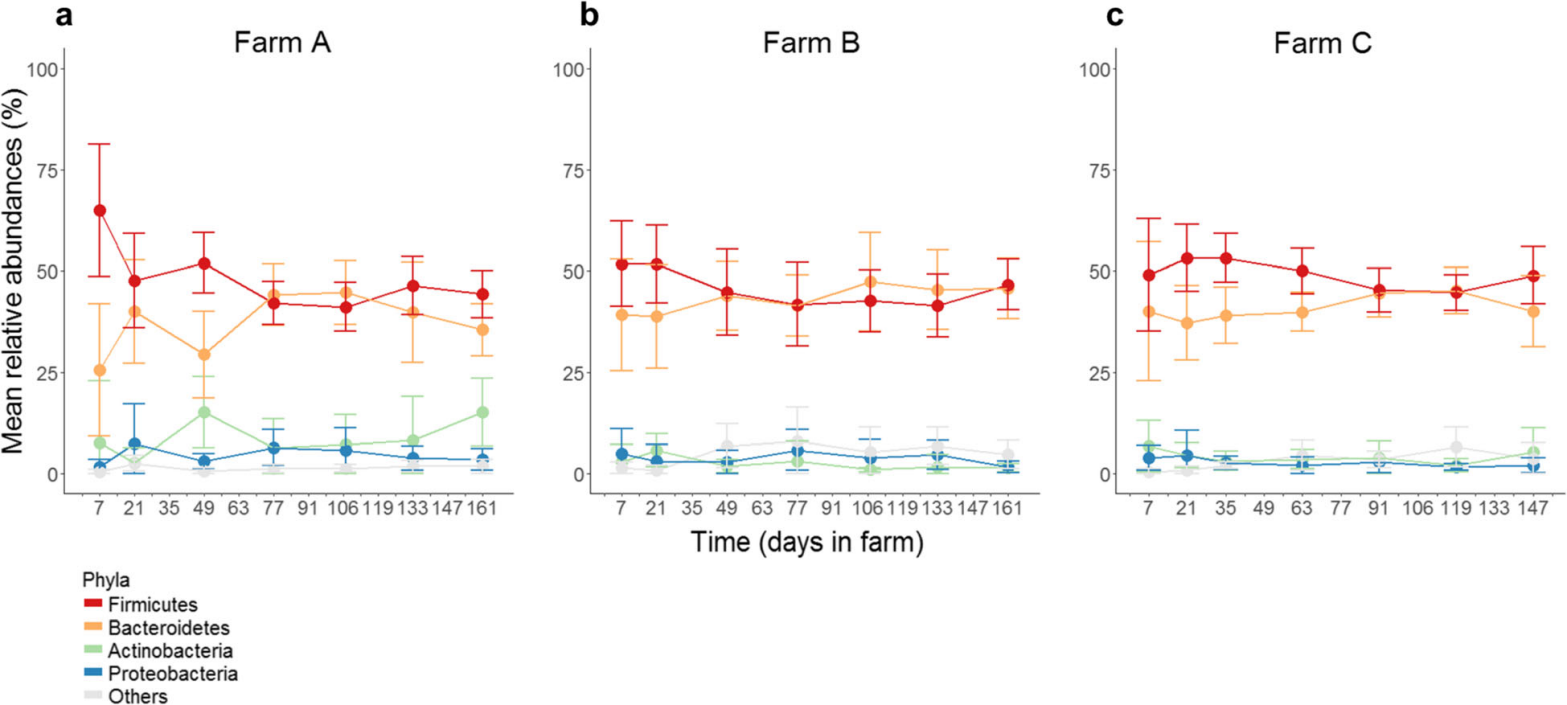
Mean relative abundance of the four main phyla over time in each farm. The mean relative abundance of the four main phyla on farm A, farm B, and farm C are represented in panels (**a**), (**b**), and (**c**), respectively. The mean relative abundances ± standard deviations of the data are represented by the bars

We investigated the taxonomic composition at different levels, from phyla to OTUs. We detected 19 phyla in the samples. Among them, only *Firmicutes*, *Bacteroidetes*, *Actinobacteria*, and *Proteobacteria* were present at a relative abundance above 1% in all samples. The phyla *Firmicutes* and *Bacteroidetes* were dominant on all farms throughout the period of the study. Their overall mean relative abundances were 47.7% (± 10.2) and 40.3% (± 11.3), respectively. The overall mean relative abundance of *Actinobacteria* was 5.2% (± 7.2) and that of *Proteobacteria* 3.7% (± 4.3). On farm A, *Firmicutes* was predominant at the beginning and then decreased slightly towards relative abundances similar to those of *Bacteroidetes* (Fig. 3).

**Fig. 4:**
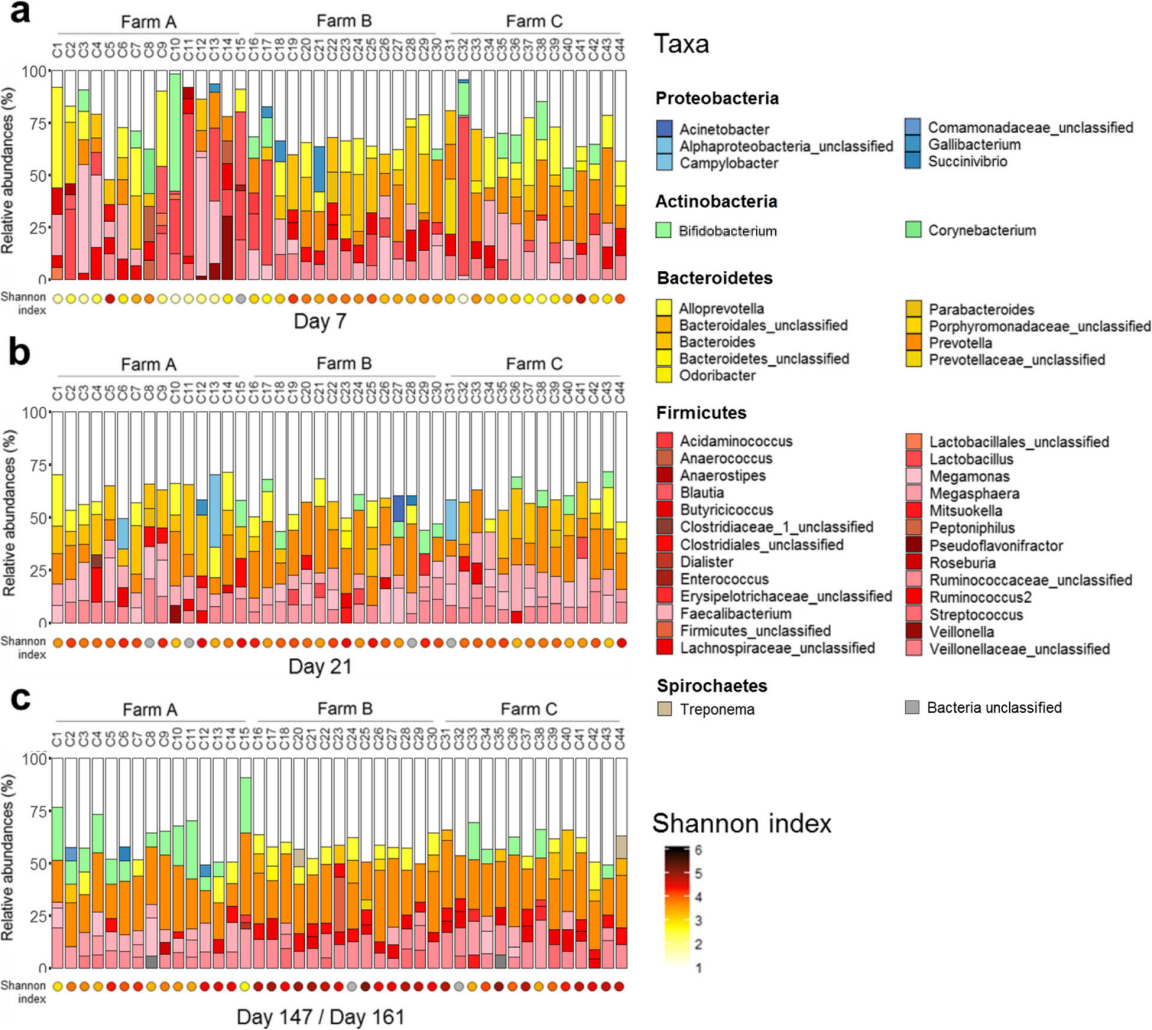
Individual microbiota composition at the genus level at the first and last month of fattening. Relative abundance of the five most abundant taxa at the genus level for all calves for the (**a**) first sampling (day 7), (**b**) second sampling (day 21), and (**c**) last sampling (day 161 for farms A and B and day 147 for farm C). Other detected taxa are depicted in white. Calf IDs are given at the top of the panels and are ordered according to farm. The color scale of the dots beneath the bar graphs represents the distribution of the Shannon index values. Grey dots indicate samples for which no index was computed because the number of sequences was lower than the rarefaction threshold. The color key refers to the phylum of each taxa and each palette was built to maximize the distinctiveness between shades

We detected 349 genera, of which 50 (14%) were found to be one of the five most abundant taxa in at least one sample (Fig. 4, Additional file 4: Fig. S4). On day 7, the mean cumulative relative abundance of the five most abundant taxa was 73.9% (± 12.3) (Fig. 4a). On day 21, the mean cumulative relative abundance of the five most abundant taxa was 59.0% (± 7.4) and it was 60.2% (± 8.4) on the last day (Fig. 4b and c).

From the second month, 16 taxa at the genus level were detected in all calves at each sampling until the end of fattening. They were *Alloprevotella, Bacteroides*, *Bifidobacterium, Blautia, Dorea, Faecalibacterium, Lactobacillus, Olsenella, Parabacteroides, Prevotella*, *Pseudoflavonifractor, Ruminococcus2,* unclassified taxa from *Clostridiales, Erysipelotrichaceae, Lachnospiraceae*, and *Ruminococcaceae*. The overall mean cumulative relative abundance of these taxa was 67.5% (± 9.9), and was similar across samplings throughout the fattening period. On farm C, the mean was equal to 74.1% (± 5.5), 72.8% (± 5.5), and 70.0% (± 6.5) on days 35, 91 and 147, respectively. On farms A and B, the mean was 64.6% (± 10.0), 64.6% (± 8.8), and 65.5% (± 7.7) on days 49, 106, and 161, respectively.

**Fig. 5:**
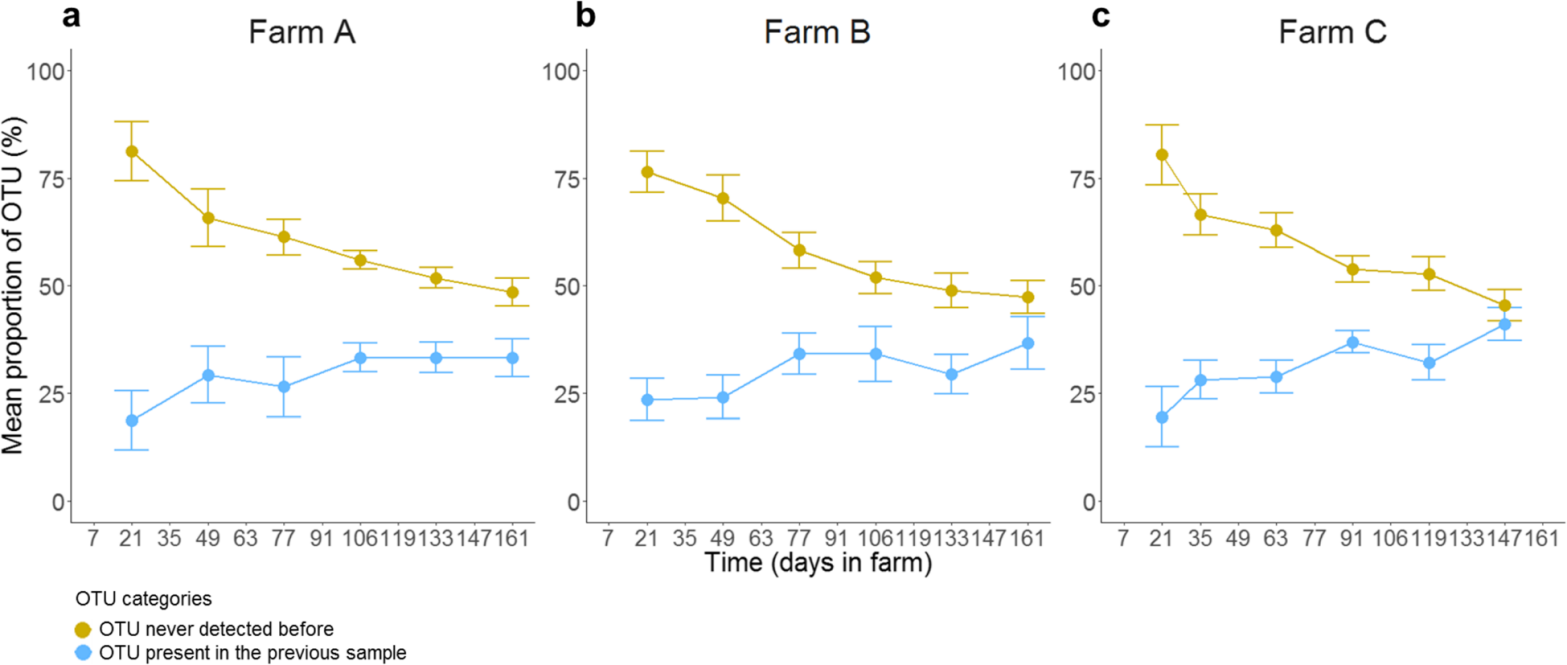
Mean proportion of OTUs relative to those detected in previous samples of the same calf. The mean proportion of OTUs for farm A, farm B, and farm C are represented in panels (**a**), (**b**), and (**c**), respectively. The mean proportions ± standard deviations of the data are represented by the bars

For each calf, the proportion of OTUs not previously detected was higher than the proportion of OTUs detected in the previous sample (Fig. 5). The proportion of OTUs detected in the previous sample varied between 7.2 and 48.6%, whereas the proportion of OTUs that had never been detected before varied between 51.4 and 92.8%. These results suggest that the temporal dynamics of the calf microbiota is driven by the replacement of autochthonous OTUs by the newly detected ones. The proportion of newly detected OTUs tended to decrease over time, concomitantly with an increase in the proportion of OTUs detected in two consecutive samples (Fig. 5). Only 50 OTUs simultaneously persisted in more than 97% of calves between consecutive samples. An OTU from the genus *Faecalibacterium* and an unclassified OTU from the *Ruminococcaceae* family persisted from the first to last month in more than 97% of calves (Additional file 5: Table S1). No OTU was newly detected or lost simultaneously by more than 50% of the calves (Additional file 5: Table S1).

No over-abundance or depletion of taxa was detected at the phylum (Fig. 3), nor genus level (Fig. 4a and b) in samples of calves under antibiotic treatment or those that had received antibiotics in the 15 days before sampling relative to calves not exposed during the same period. For example, at day 21, although calves on farm A had received antibiotics for 20 days while calves on farm B had not, 154 genera were detected on both farms. They represented 86.5 and 85.6% of the genera detected that day on farms A and B, respectively. At day 21, 157 genera were detected on both farms B and C. They represented 87.2 and 86.7% of the genera detected that day on farms B and C, respectively, despite the fact that calves on farm C had received antibiotics for 14 days. Sixteen taxa at the genus level were only detected on farm B and were found at a relative abundance of < 1% (*Chlamydophila, Snodgrassella,* unclassified taxa from *Rhodobacteraceae, Tissierella, Clostridium_XI, Comamonas, Basfia, Janibacter, Anaerosporobacter, Pseudoscardovia,* unclassified taxa from *Deltaproteobacteria, Brevinema, Brucella,* unclassified taxa from *Gammaproteobacteria,* unclassified taxa from *Desulfovibrionaceae, Oligosphaera).*

### Shannon index and the number of observed OTUs

**Fig. 6:**
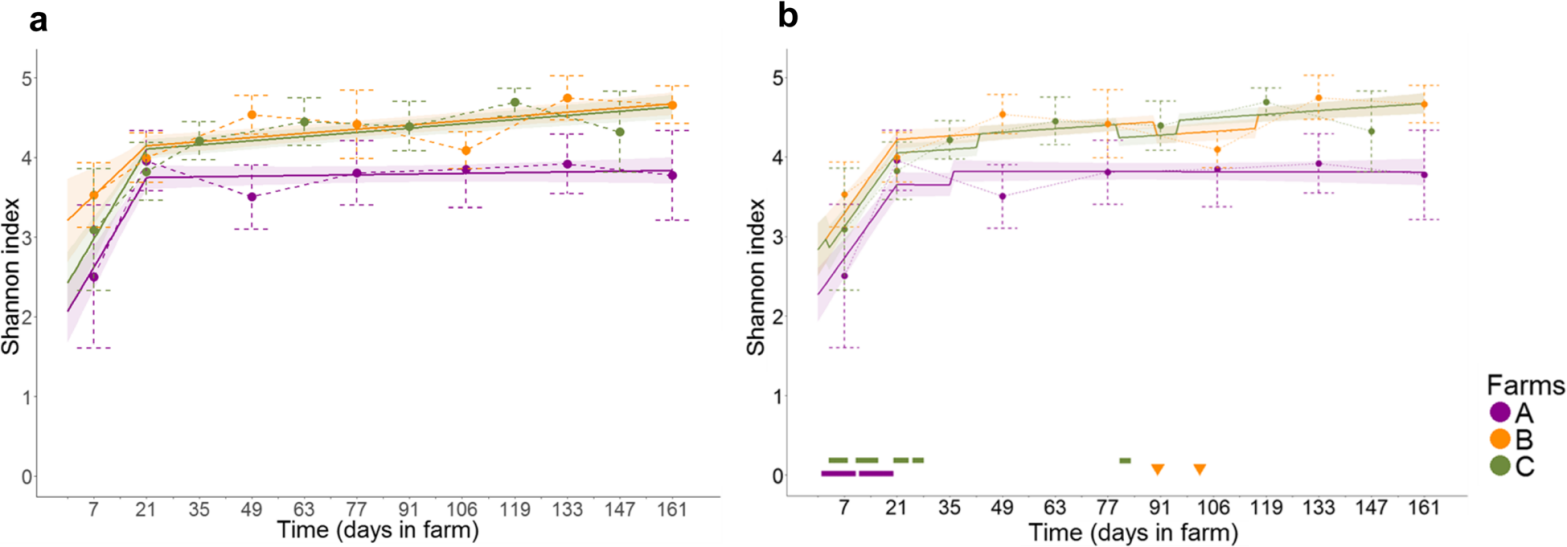
Dynamics of the mean observed and predicted Shannon index for each farm. The predicted dynamics of the Shannon index, without and with the antibiotic-treatment effect, in the final model are shown in panels (**a**) and (**b**), respectively. The mean values ± standard deviations of the observed data for each farm are represented by the dashed bars. Model-predicted profiles and their 95% confidence bands are represented by the solid lines and bands, respectively. Antibiotic treatments during sampling or within 15 days before sampling are colored coded by farm and indicated above the x-axis in panel (**b**)

We evaluated the temporal dynamics of microbiota diversity in each sample by examining the α-diversity metrics, the Shannon index, and the number of observed OTUs. The Shannon index showed an increasing trend over time, with an overall mean of 3.05 (± 0.82) on day 7 and 4.23 (± 0.59) at the end of fattening. The temporal dynamics of microbiota diversity were best described by a two-slope model (Likelihood Ratio Test (LRT) between the two candidate models, *p* < 10^− 15^, Fig. 6a, Additional file 6: Table S2). The coefficient of the first slope was higher than that of the second, suggesting a large increase in diversity during the first month, followed by a lowering of the rate of increase, and even stagnation on farm A. The estimates for the intercept and second slope for farms B and C were significantly higher than those for farm A. There was a similar temporal trend and a significant farm effect on the number of observed OTUs (Additional file 7: Fig. S5a, Additional file 6: Table S3).

A variable representing antibiotic treatment in the 15 days previous to sampling (still ongoing or not at the time of sampling) was added to the two-slope model and tested for significance for both indices. There was a significant effect of antibiotics on the Shannon index intercept, with an estimated decrease of − 0.17 CI_95%_[− 0.27; − 0.06] (*p* = 0.003, Additional file 6: Table S2, Fig. 6b), indicating an antibiotic-induced reduction of bacterial diversity during exposure. The effect of antibiotic exposure on the number of observed OTUs was also significant and showed a similar effect of decreasing diversity, with an estimated decrease of 82.7 OTUs CI_95%_[− 115.8; − 49.6] (*p* = 1*10^− 6^, Additional file 6: Table S3, Additional file 7: Fig. S5b).

### Absolute number of *E. coli* per gram of feces

**Fig. 7:**
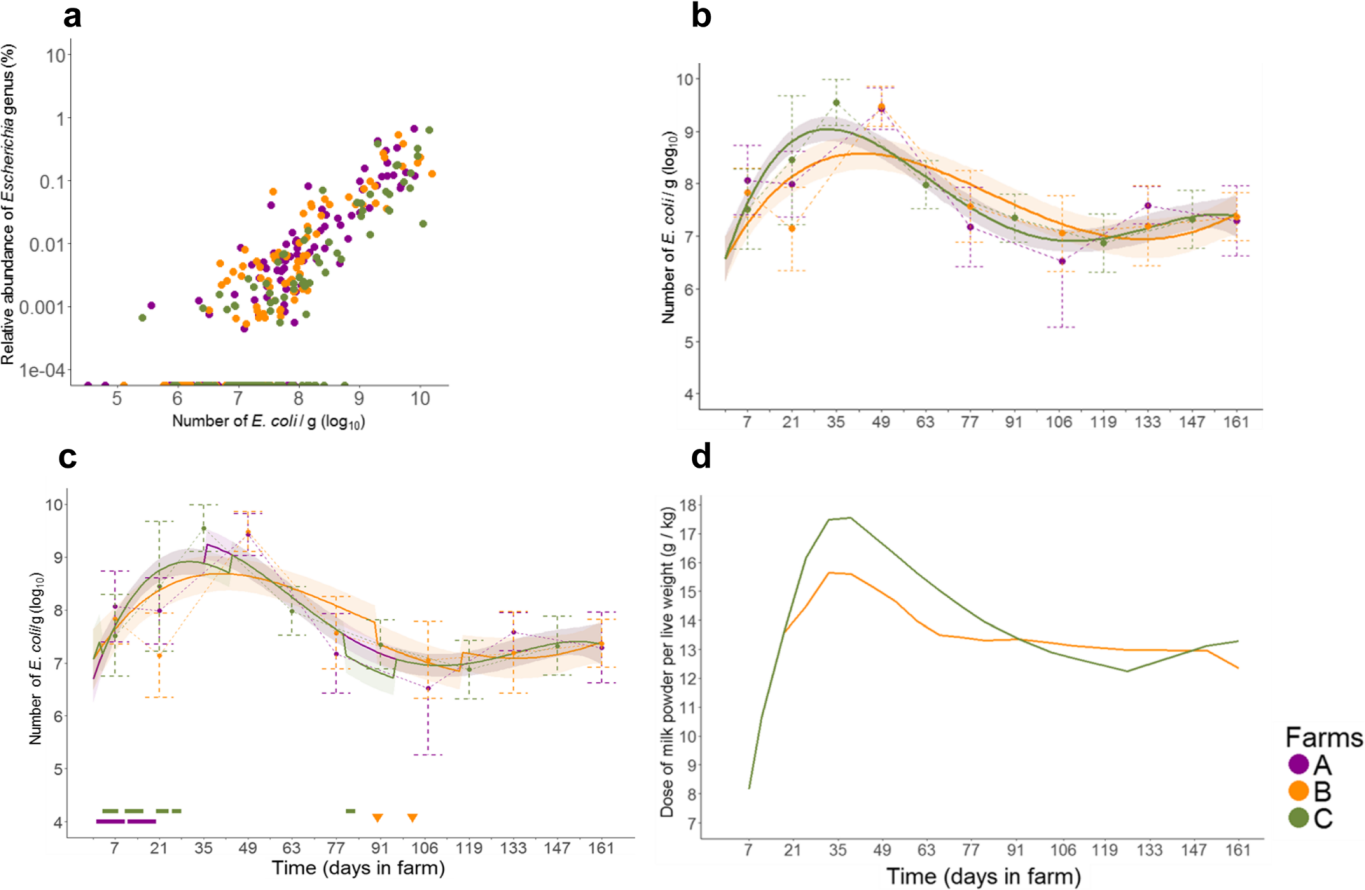
Absolute quantification of the *Escherichia coli* population by qPCR. **a** Relative abundance of the *Escherichia* genus as a function of the number of *E. coli*/g estimated by qPCR. Each point represents a sample. Points on the x-axis represent samples for which no 16S rRNA gene sequence of the *Escherichia* genus was detected by sequencing. **b** Dynamics of the mean observed and predicted number of *E. coli*/g for each farm in the final model without the antibiotic-treatment effect. The mean values ± standard deviations of the observed data for each farm at each sampling time are represented by the dashed bars. The model-predicted profiles and their 95% confidence bands are represented by the solid lines and bands, respectively. The predicted profiles of farms A and C are overlapping, which is why the predicted profile of farm A does not appear. **c** Dynamics of the mean observed and predicted number of *E. coli*/g for each farm in the final model with the antibiotic-treatment effect. Antibiotic treatments during sampling or within 15 days before sampling are color-coded by farm and indicated above the x-axis. **d** Temporal dynamics of the recommended dose of milk powder per kilo of live weight for farms B and C

We quantified the absolute number of *Escherichia*/g by qPCR, which can be considered as a fair proxy of the absolute number of *E. coli* in calves' feces (see Methods). *E. coli* is the main facultative anaerobic bacteria in the large intestine and a marker of dysbiosis [12]. We first compared these absolute numbers to the relative abundance of the *Escherichia* genus estimated by 16S rRNA gene sequencing. The number of *E. coli* estimated by qPCR was strongly and positively associated with the relative abundance of the *Escherichia* genus (Spearman’s correlation, r_s_ = 0.80, *p* < 10^− 15^, Fig. 7a), confirming the strong relationship between the two variables. The temporal dynamics of the number of *E. coli*/g was similar for the three farms, with an overall mean of 7.81 log_10_ (*E. coli*/g) (± 0.67) on day 7. During the second month, a transient but important increase (approximately 2 log_10_) occurred on the three farms (Fig. 7b). The dynamics of the number of *E. coli*/g were best described by a quartic function of time (LRT between cubic and quartic function of time models, *p* = 0.01). The final model gave the same estimates for the parameters of farms A and C, suggesting the presence of an additional factor in shaping the number of *E. coli*/g on these farms (Fig. 7b and Additional file 6: Table S4).

As for α-diversity indices, a variable representing antibiotic treatment in the 15 days prior to sampling (still ongoing or not at the time of sampling) was added to the quartic model and tested for significance. There was a significant reduction in the *E. coli* population in calves treated in the previous 15 days relative to those that were not, with an estimated decrease of − 0.37 log_10_ (*E. coli*/g) CI_95%_[− 0.66; − 0.08] (*p* = 0.01, Additional file 6: Table S4, Fig. 7c).

### Association between the estimated dose of milk powder given in two farms and bacterial abundance

In farms B and C, for which the information was available, we explored associations between the relative abundance of genera and the daily dose of milk powder recommended by the integrator. Calves were almost exclusively fed milk replacer throughout the fattening period, which is reconstituted from milk powder with hot water. Their diet was also supplemented with a small amount of solid feed from the first weeks. We conducted 349 Spearman rank correlation tests and found 17 taxa at the genus level for which the Spearman correlation coefficient was positive and significantly different from zero (Table 1, Additional file 8: Table S5). The genus with the strongest correlation was *Megasphaera*, with a moderate to strong association (r_s_ = 0.60, Bonferroni adjusted *p* < 1*10^− 15^, Table 1, Additional file 9: Fig. S6). The genera with the next highest positive correlation coefficients were *Enterococcus*, *Dialister*, and *Mitsuokella,* indicating moderate association with the dose of milk powder (r_s_ = 0.44, 0.42, and 0.41, and Bonferroni adjusted *p* = 2*10^− 8^, 3*10^− 7^, and 6*10^− 7^, respectively, Table 1, Additional file 9: Fig. S6). The genus *Escherichia* was also positively associated with milk powder, but to a lesser extent (r_s_ = 0.28, Bonferroni adjusted *p* = 0.02, Table 1).

**Table 1:** Correlation between the dose of milk powder and fecal microbiota on farms B and C

Taxa identified at the genus level*	Spearman correlation coefficient^a^	*p-*value	Bonferroni adjusted *p-*value
*Megasphaera*	0.60	2*10^−20^	6*10^−18^
*Enterococcus*	0.44	5*10^−11^	2*10^−8^
*Dialister*	0.42	1*10^−9^	3*10^−7^
*Mitsuokella*	0.41	2*10^−9^	6*10^−7^
*Collinsella*	0.37	9*10^−8^	3*10^−5^
Unclassified *Veillonellaceae*	0.32	4*10^−6^	0.001
*Sutterella*	0.31	1*10^−5^	0.003
*Selenomonas*	0.31	1*10^−5^	0.003
*Anaerofilum*	0.30	2*10^−5^	0.006
*Faecalibacterium*	0.30	2*10^−5^	0.007
*Butyricimonas*	0.29	4*10^−5^	0.01
*Megamonas*	0.28	6*10^−5^	0.02
*Escherichia/Shigella*	0.28	8*10^−5^	0.02
*Fastidiosipila*	0.27	0.00013	0.038
*Helcococcus*	0.27	0.00014	0.040
*Bifidobacterium*	0.27	0.00014	0.041
*Psychrobacter*	0.27	0.00015	0.045

* Data represent the taxa identified at the genus level with a positive correlation with the dose of milk powder estimated on farms B and C. Only taxa with a significant correlation (Bonferroni corrected *p-*value < 0.05) are shown

^a^ Spearman correlation coefficients ∈ [0.4; 0.6] indicate moderate to strong associations, whereas coefficients < 0.4 indicate weak associations and were not considered for further analyses

As *E. coli* is a β-galactosidase-positive species, which means it is able to cleave lactose into monosaccharides, we compared the estimated dynamic profiles of the absolute number of *E. coli*/g and the dose of milk powder recommended by the integrators on farms B and C. The estimated profiles were very close for both farms, particularly during the second month, with the peak of the dose of milk powder superimposed over that for the number of *E. coli*/g (Fig. 7d). We searched for an association between the estimated daily doses of milk powder and the farm predicted numbers of *E. coli*/g on farms B and C using Spearman’s correlation test. We found a significant strong positive association between the farm predicted numbers of *E. coli*/g and the estimated dose of milk powder (r_s_ = 0.77, *p* < 1*10^−15^).

## Discussion

We characterized the dynamics of the fecal microbiota of calves from two weeks to six months of age on three commercial veal farms representative of the three main French integrators and of management practices in the veal industry in France. The calves were mainly fed milk replacers throughout the follow-up and received several collective antibiotic treatments at therapeutic doses, most administered in the first weeks of fattening (Fig. 1). We performed 16S rRNA gene sequencing to study the composition of the microbiota and qPCR of the *Escherichia* genus as a proxy of *E. coli* to quantify its commensal populations. We estimated the daily dose of milk powder recommended by the integrator for two farms to search for an association with the relative abundance of the detected genera. The most striking results of this study are (i) the convergence of the fecal microbiota composition among calves, which began during the first month of life, along with an increase in α-diversity, (ii) a decrease in microbiota diversity and the size of the *E. coli* population during or within the 15 days following an antibiotic treatment relative to non-exposed calves of the same age (reduction of the Shannon index by 0.17 and the number of *E. coli*/g of feces by 0.37 log_10_ (*E. coli* / g)), and (iii) a significant association between the estimated daily dose of milk powder and the relative abundance of four genera (*Megasphaera*, *Enterococcus*, *Dialister*, *Mitsuokella)* and the predicted farm profiles of the number of *E. coli*/g from our model.

The development of the microbiota of these calves was characterized by the dominance of a small number of genera mainly from the *Firmicutes* and *Bacteroidetes* phyla (Fig. 3). These phyla remained dominant as the calves aged (Fig. 4), with a simultaneous increase in microbiota diversity (Fig. 6 and Additional file 7: Fig. S5a). These developmental features have already been described for calves with the same characteristics (age, sex, and breed) fed milk replacers [10], as well as for females of the same breed in Canada [10, 13], the USA [14, 15], and Japan [16] and for dairy calves of a different breed in Austria [17]. These shared findings suggest that the fecal microbiota of calves undergoes a predictable age-dependent trend that is common to distinct calf populations.

The high heterogeneity of the microbiota composition at day 7 on the farms (corresponding to 3 weeks of age) is likely attributable to the distinct origin of the calves, as they came from different dairy farms. Prior studies have noted the important influence of exposure to the bacterial communities of both the dam and the environment on the composition of the microbiota throughout the gut of the newly born calf [18, 19]. The transport from dairy farms to veal farms could also be responsible for the high heterogeneity between calves at the beginning of fattening. Transport has been reported to have disrupting effect on the gut microbiota in young beef cattle 5 days after their transport to the feedlot [6]. The composition of milk replacers may have been a source of variability between farms during fattening. A difference in the relative abundance of *Bifidobacterium* spp. and *Faecalibacterium prausnitzii* was found between one-month-old dairy calves fed milk replacers containing different levels of proteins and fat, although this difference was transitory [20].

From as soon as the end of the first month after arriving on the farms, bacterial succession (Fig. 5) gradually increased the similarity of the microbiota composition among the calves on all farms. This increase was due to both an increase in sharing of the same bacterial members and the homogeneity of their relative abundance, as shown by unweighted and weighted Unifrac distances (Additional file 2: Fig. S2 and Fig. 2, respectively). This convergent pattern occurred in the absence of environmental or dietary changes, such as weaning, the calves being reared in dedicated closed buildings and drinking milk replacers throughout fattening. This suggests that the influence of environmental and dietary factors on such convergence was probably limited and highlights the likely role of host physiology. Convergence related to age has also been observed in the ruminal microbiota of calves between one day and two years of age [21] and in both the ruminal and fecal microbiota of dairy heifers receiving different diets before weaning [22]. These studies suggest that such changes in composition are not restricted to the lower part of the gut and are not strongly driven by diet. An intriguing possibility is that such convergent stabilization of the microbiota composition over time (Fig. 2, Additional file 2: Fig. S2) may be linked to age-dependent shifts of the gut mucosal immune system, as the expression of Toll-like receptors in both the rumen and colon have been shown to change as calves age [23]. Constraints imposed by the gut environment and autochthonous microbiota on allochthonous bacterial settlement may become less permissive, resulting in more specific requirements as the calves aged.

The microbiota of calves on farms where collective antibiotic treatments were given in the previous 15 days or during sampling underwent a reduction in diversity and the number of *E. coli* relative to calves of the same age that had not been exposed during the same period (Fig. 6b, Additional file 7: Fig. S5, Fig. 7c). We pooled the effects of both long-term and short-term antibiotic treatment and molecules with different spectra to focus on the common disruptive effects of antibiotics on microbial ecosystems. As all calves of the same farm received the same antibiotic treatment at the same time, the design of our study did not allow the analysis of the specific effects of each molecule. As the treatments were collective, we did not have negative controls for calves receiving antibiotics from the same farm. Hence, the results could have been biased by farm-specific variables, which could have modulated the relative abundance of certain genera. To our knowledge, no study has yet explored the impact of antibiotic treatments on the maturation of gut microbiota in veal calves. Moreover, literature on the effect of each antibiotic class on the hindgut microbiota of preweaned dairy calves is scarce. As far as we know, research on this topic has focused on a few classes: macrolides [7, 24], tetracycline [7], amphenicol [7], fluoroquinolones [24], and polypeptides [25]. These studies, with the exception of that by Xie et al. [25], all focused on the effects of a single injection of the antibiotic, which differs from the treatment plans and route of administration (oral) in our study. Xie et al. reported dysbiosis in neonatal dairy calves receiving bacitracin for 10 days in the milk replacer, characterized by an increase in the abundance of *Escherichia* and *Enterococcus*, along with a decrease in the abundance of *Dorea, Collinsella, Eubacterium, Faecalibacterium, Papillibacter, Peptostreptococcus*, *Prevotella,* and *Roseburia,* relative to non-exposed calves of the same age [25]. The modalities of treatment in this study were close to those for the calves of farms A and C just after their arrival on farms. On farm A, they received a combination of antibiotics in their feed for several days, including colistin, another polypeptide. Their finding concerning the genus *Escherichia* is contrary to the negative effect of antibiotics that we found. This may be explained by the difference of the spectrum between bacitracin and the antibiotics used on the veal farms, as bacitracin has a narrow spectrum, targeting Gram-positive bacteria, whereas *E. coli* is sensitive to antibiotics used during extended treatment (colistin, sulfonamides, tetracycline, trimethoprim). It may also be partially explained by the age of calves, as the microbiota change markedly between birth and the first month of life [16, 26].

Antibiotic-induced loss of diversity has already been reported in young beef cattle [6], as well as pre-weaned calves [7], and was often found to be associated with the depletion of beneficial bacteria and/or the increase of opportunistic pathogens [7, 25]. Antibiotic-induced dysbiosis is also observed when pre-weaned calves are fed low doses of antibiotic molecules [27].

The effect of the antibiotic treatments was small relative to the longitudinal changes (Additional file 6: Tables S2, S3 and S4), consistent with findings in pre-weaned dairy calves receiving enrofloxacin and tulathromycin metaphylactic treatment [24] and beef cattle receiving oxytetracycline or tulathromycin injection [6]. These findings can be explained by the existence of a natural resistome, independently of any antibiotic treatment, carried by certain abundant families in the fecal microbiota of pre-weaned calves, as recently shown [28]. Genera of these antibiotic-resistance gene carrying families were found to be dominant in the feces of the veal calves, such as *Anaerostipes, Blautia,* and *Roseburia* (*Lachnospiraceae* family), *Enterococcus* (*Enterococcaceae* family), *Faecalibacterium* and *Pseudoflavonifractor* (*Ruminococcaceae* family), *Bacteroides* (Bacteroidaceae family), and *Streptococcus* (*Streptococcaceae*). Members of the *Enterobacteriaceae* family, such as *E. coli,* were also found to be a major reservoir of antibiotic-resistance genes within the microbiota resistome.

Two recent studies reported that the resistome of fecal microbiota in pre-weaned dairy calves is composed of resistance-conferring genes against tetracycline, sulfonamides, trimethoprim, β-lactams, and macrolides [8, 28]. These results suggest the existence of a natural resilience in fecal bacterial communities to collective antibiotic treatments on veal farms; the antibiotics used to treat the calves in our study belonged to these classes. None of these studies reported the presence of the *mcr-1* gene, which confers resistance to colistin, another molecule used to treat calves, in microbial communities, although another study detected the gene in the commensal *E. coli* of veal calves [3]. Moreover, it has been shown that this natural resistome is shaped by the bacterial phylogeny of the fecal microbiome and decreases as the calves age. One of the main drivers of such a decrease was the decrease in abundance of the *Enterobacteriaceae* family, in which 90% of the members were classified as *E. coli* [28]. It is well-known that commensal *E. coli* populations of veal calves harbor high levels of antibiotic-resistance genes [3, 29], and that they are diverse. Antibiotic treatment may have promoted an increase in the number of specific pre-existing *E. coli* strains at the beginning of fattening, as the extended treatments during the first month did not result in marked depletion of the *E. coli* population.

Among others, we found a possible link between *E. coli* population dynamics and the use of milk replacer, which is reconstituted from dry milk powder and rich in lactose (Fig. 7d) [30]. Lactose allows the growth of the vast majority of *E. coli* strains [31]. It has been shown that the lag time and generation time of *E. coli* strains, which depend on metabolic efficiency and are crucial for gut colonization and persistence [32, 33], are influenced by the type and abundance of available nutrients in the habitat [34]. Other components of milk replacers may have also influenced the *E. coli* population dynamics, such as vitamin D, for which the absence of the vitamin D receptor in the intestinal epithelium of mice has been associated with increased *E. coli* loads [35]. Our findings are consistent with those of another study in which the fecal microbiota of Simmental calves was followed during their first 3 months of life, although only six calves were included in the study and the sampling was sparse [17]. The relative abundance of the genus *Escherichia* was found to be maximal during the milk-feeding period and decreased before weaning.

Lactose, which is one of the main components of milk powder (approximately 45% of the dry weight), is hydrolyzed to the monosaccharides glucose and galactose by bacteria that synthesize the β-galactosidase enzyme. We therefore looked for the presence of this enzyme in available annotated genomes from the NCBI genome database of the *Megasphaera*, *Enterococcus*, *Dialister*, and *Mitsuokella* genera. The sequence of *E. coli* LacZ β-galactosidase was also compared to the protein sequences found in members of these genera using the blastp program [36, 37]. A β-galactosidase gene sequence was found in the genome of *Mitsuokella multacida*, which was isolated from human feces [38], and several species of the *Enterococcus* genus, which have been isolated from cattle (*E. faecalis, E. faecium, E. hirae, E. thailandicus, E. malodoratus, E. devriesei, E. casseliflavus, E. italicus*) [39–42] (data not shown). The concomitant fluctuations of this lactose-rich source and the relative abundance of the genera *Enterococcus* and *Mitsuokella* strongly suggest a direct role of host diet on members of the fecal microbiota. We did not find a β-galactosidase annotated gene in the 24 *Megasphaera* annotated genomes nor in the 18 *Dialister* annotated genomes available on NCBI [37]. The bacteria of these two genera did not carry a protein similar to the *E. coli* LacZ β-galactosidase (data not shown). The discrepancy between the associations found for the genera *Megasphaera* and *Dialister* and the absence of the β-galactosidase enzyme sequence in the genome of known members of these genera could be explained by the utilization of another nutrient present in the milk powder by the members of these taxa. Another explanation for this discrepancy could be the limited redundancy of carbon source use among members of these genera coupled with a small number of genomes from these genera in the NCBI database.

Our study had several limitations. First, we followed the fecal microbiota of calves reared in commercial veal farms. Thus, the calves were not randomly assigned to the different farms (nor antibiotic treatments) and neither the environmental nor dietary variables could be controlled as they would be in a randomized trial. Nevertheless, one of the aims of this study was to characterize the fecal microbiota of calves reared following common veal-farm practices. These three farms, in which field studies had already been conducted [3], are representative of management practices in the veal-calf industry in France. Second, the calves were only sampled after spending 7 days on the farms. Thus, no information in terms of their microbiota composition before antibiotic treatment was available. They were also sampled on a monthly basis, whereas high-frequency sampling has been recommended in early-life microbiome studies in infants [43]. Furthermore, sampling was performed independently of antibiotic treatment. Hence, certain short- and mid-term age- and antibiotic-associated changes may have been missed. Third, although we tracked the dynamics of microbiota using 16S rRNA gene sequencing and *E. coli* qPCR, we only focused on specific features of this complex ecosystem and may have missed specific patterns at other levels. For example, we had no information concerning the dynamics of fecal bacterial loads, which have been shown to vary in newborn calves [9, 44, 45] and be associated with microbiota composition [46].

Nevertheless, veal calves, as studied here, have attributes relevant to the exploration of the microbiota maturation process. First, batches are composed of male calves of the same age and breed (usually Holsteins). Thus, they are highly genetically and physiologically homogeneous. Second, they share the same living environment and diet, which are not subject to major changes, as they are reared in dedicated closed buildings in which the conditions are stable and controlled to optimize their growth. Moreover, they do not experience any drastic changes in their diet, as it remains predominantly composed of milk replacers during the 6 months of fattening. Third, the systematic administration of antibiotics at therapeutic doses to all members of the batch is common practice to prevent the spread of infectious diseases [2]. As healthy young subjects that share similar controlled conditions over a long period of time and experience common antibiotic exposure, veal calves represent a unique opportunity to disentangle the factors that drive microbiota assembly under real-life conditions.

## Conclusion

This observational study conducted on calves reared under intensive-farming conditions shows (i) early convergence of the developing fecal microbiota among farms and (ii) a significant association between the estimated daily doses of milk powder and the relative abundance of certain genera and the predicted farm profiles of the number of *E. coli*/g. This study also suggests that the administration of collective antibiotic treatment results in a limited reduction of diversity and size of the *E. coli* population and highlights the need for additional studies to fully understand the impact of antibiotic treatment in the context of the veal industry. To our knowledge, this is the first field study to follow the microbiota composition and size of the commensal *E. coli* population of veal calves throughout the fattening period.

## Methods

### Animal handling and sampling

We collected fecal samples from veal calves during a cohort study dedicated to monitor the excretion of extended spectrum β-lactamase- (ESBL)-producing *E. coli*, which has been shown to be frequent in veal calves [3, 47]. The fecal excretion of ESBL-producing *E. coli* was followed in 45 veal calves distributed in three French veal farms (named A, B, and C) located in the region of Brittany [3], within a 100-km radius around Rennes. The three farmers raised calves in partnership with different integrators, which were the main veal calves producers in France. We streaked swabs on selective ChromID ESBL agar (bioMérieux, Marcy l’Etoile, France) and classified calves as “ESBL-producing *E. coli* high-level excretor”, “low level excretor” or “ESBL-producing *E. coli*-free” based on the number of colonies that grew after 24 h at 37 °C (> 100 colonies = high level excretion, < 100 colonies = low excretion, or no colonies = no excretion) [3]. This cohort also provided an opportunity to follow the dynamics of the fecal microbiota of veal calves under real-life conditions by the collection of additional fecal samples. Characterization of the ESBL-producing *E. coli* will be published elsewhere.

Sampling began upon the arrival of batches of new calves in October and November 2015. A batch was defined in the study as a group of calves entering the farm at the same time and reared together until slaughter (Additional file 10: Fig. S7). Seven days after arrival, 15 calves were randomly selected from 50 on each farm and included in the study: five with high levels of ESBL-producing *E. coli* excretion, five with low-level excretion, and five with no excretion. The 15 calves selected per farm were then sampled by rectal swabbing bimonthly for ESBL-producing *E. coli* excretion follow-up until departure of the batch to the slaughterhouse. To study the dynamics of the fecal microbiota, additional samples were collected at days 7 and 21 on the farms, then monthly for 5 months, for a total of seven samples per calf (Fig. 1). Swabs were placed immediately in portable coolers with ice packs, shipped to the ANSES lab in Lyon, France, and stored at − 80 °C.

The calves were 14 days old when they arrived at the farms and were mainly fed milk replacer, which is reconstituted from cow milk powder with hot water, throughout fattening. Their diet was also supplemented with a small amount of solid feed from the first weeks. The daily quantity of milk powder was divided by the mean weight of a male Holstein calf of the corresponding age each day (assuming that the calves arrived at the farms at 14 days of age), which enabled us to estimate a proxy for the dose of milk powder consumed on these farms throughout the fattening period. Thus, the integrators’ recommended doses of milk powder, of which approximately 45% consists of lactose, were estimated for farms B and C.

Collective antibiotic treatments were recorded by the three farms throughout fattening (Fig. 1). Antibiotics were always used at therapeutic doses and administered orally in water or milk replacer. All treatments upon entry to the fattening farms were set up to prevent gastrointestinal disorders, whereas treatments during the course of fattening were used to treat respiratory diseases. All calves received antibiotics more than once and calves from farms A and C received several consecutive antibiotic treatments during the first month (Fig. 1). On farm A, calves received a 10-day course of colistin and sulfonamides (first day to day 10), followed by another 10-day course of tetracycline (day 11 to day 20). Later during fattening, they received a one-day treatment of doxycycline on day 53 and a one-day treatment of amoxicillin on day 135. On farm B, calves received a one-day treatment of doxycycline and erythromycin on day 26, a one-day treatment of tetracycline on day 90, and a one-day treatment of amoxicillin on day 101. On farm C, calves received a six-day course of sulfonamides and trimethoprim (from day 3 to day 8) and a seven-day course of tetracycline (day 10 to day 16). They also received a five-day course of doxycycline (day 20 to day 24) and four-day courses of spiramycin (day 25 to day 28) and tetracycline (day 80 to day 83).

### DNA extraction, 16S rRNA gene sequencing, and *Escherichia-*specific quantitative PCR

Genomic DNA was extracted from rectal swabs using the DNEasy PowerSoil kit (QIAGEN, Venlo, Netherlands). The cotton tips of frozen swabs were broken off directly into bead tubes. The tubes were incubated at 70 °C for 10 min, as previously described [48]. The remaining steps were performed according to the manufacturer’s instructions, with an additional overnight incubation step with elution buffer at 4 °C. Extracted DNA was stored at − 20 °C. The V4 region of the 16S rRNA gene from each sample was amplified using the primers 515fB (GTGYCAGCMGCCGCGGTAA) and 806rB (GGACTACNVGGGTWTCTAAT), modified to contain a barcode sequence between the primer and Illumina adaptor sequences, as previously described [49, 50]. Dual-barcoded libraries were sequenced on an Illumina MiSeq machine (MiSeq Reagent Kit V3, 600 cycles) according to the manufacturer’s specifications to generate paired-end reads of 300 bases in length.

*E. coli* populations were quantified by qPCR targeting the 16S rRNA gene sequence specific to the *Escherichia* genus. The *Escherichia* genus is a good proxy of *E. coli* in cattle, as *Escherichia* cryptic clades and other species of the genus represent < 4% in non-human mammal feces [51]. We verified this assumption by plating three randomly selected swabs from distinct calves of our study on Drigalski plates. We determined the species of 50 colonies per plate using MALDI-TOF and Clermont typing PCR [52]. All colonies were confirmed to be *E. coli*. The sequences of the forward (CATGCCGCGTGTATGAAGAA) and reverse (CGGGTAACGTCAATGAGCAAA) primers and probe (FAM-TATTAACTTTACTCCCTTCCTCCCCGCTGAA-TAMRA) were obtained from [53]. Each DNA sample (approximately 20 ng) was added to 30 μl PCR mixture containing 15 μl TaqMan Universal PCR master mix II 2X (Applied Biosystems, Life Technologies, Carlsbad, California, USA), 300 nM of each primer, 100 nM fluorescent probe, and bovine serum albumin at a final concentration of 0.1 μg/μl (New England BioLabs, Evry, France), as previously described [54]. A standard curve was generated using known amounts of DNA of the archetypal ED1a *E. coli* strain for each experiment. Products were detected using an Applied Biosystems Prism 7500 instrument.

### Processing of 16S rRNA gene sequences

The quality of the reads was verified using FASTQC [55] and they were processed using mothur (version 1.35.1) [56, 57]. Contigs were generated by assembling forward and reverse reads. Low-quality contigs were discarded if the total length was outside 289 to 292 bases, if there were more than five ambiguous bases (“N”), or if homopolymer runs exceeded five bases. After a clean-up step, sequences were aligned to those of the SILVA reference database (February 2017, release 128) [58]. OTU assignment was made after clustering the sequences with a similarity cutoff of 97%. Singletons, duplicates, and triplicates were discarded. The taxonomy of each detected OTU was obtained using the RDP quality-controlled, aligned, and annotated Bacterial and Archaeal 16S rRNA gene sequence database [59]. Chimeric sequences were removed after de novo chimera detection using the VSEARCH tool, version 2.3.4 [60]. Sequences flagged as chloroplasts, mitochondria, or eukaryotes were discarded from the dataset. We assessed the taxonomic composition of the microbiota, focusing on the phyla and genera, and focused on the five most abundant genera in each sample. For each calf, the OTUs detected in a sample were classified according to their detection in the previous sample to determine the monthly degree of change of the calf microbiota at the OTU level. For each time point, we determined the proportion of OTUs that were not previously detected and the proportion of OTUs that were not detected in the previous sample. This analysis was performed to identify concurrent patterns of acquisition and persistence of OTUs among calves.

A rarefaction step was performed before computation of the α- and β-diversity metrics. Several candidate rarefaction thresholds were assessed for several characteristics, such as the number of samples below the threshold, the number of samples for which the sampling effort would result in more than 25% and 50% of the total number of sequences, and the proportion of sequences sampled in the most abundant sequence sample. The threshold was set to 47,000 sequences, which was the best compromise between these four characteristics. Then, α-diversity and β-diversity metrics were computed from the rarefied samples. α-diversity is defined as the ecological diversity within samples, whereas β-diversity is defined as the dissimilarity between samples. The Shannon diversity index and the number of observed OTUs were computed as α-diversity metrics. Unweighted and raw-weighted Unifrac distances were computed as β-diversity metrics [61]. For each calf, distances were computed between consecutive samples, and the distances between all calves were computed at the first, second, and last sampling.

In summary, the available data for each sample were (i) the relative abundance of taxa at the phylum and genus level, (ii) the α-diversity metrics Shannon index and the number of observed OTUs, (iii) the weighted and unweighted Unifrac distances to the previous and next sample for the same calf, and (iv) the absolute number of *E. coli* per gram of feces. In addition, Unifrac distances were computed at the first, second, and last sampling between calves.

### Statistical analyses

#### Comparing community structures between and within calves over time

We performed PERMANOVA tests [62] using weighted and unweighted Unifrac distance matrices to evaluate the effects of time and the farm on the calf’s microbiota. Tests were performed using 1000 permutations. We constrained the permutations within each calf to account for repeated measures. The weighted and unweighted Unifrac distances between calves at the first, second, and last samplings were represented in heatmaps. Moreover, the Unifrac distances between consecutive samples for each calf were represented by spaghetti plots to assess the temporal variability of its microbiota composition.

#### Temporal modelling of α-diversity indices

We built linear mixed-effects models to study the temporal dynamics of microbiota diversity. One-slope and two-slope models were tested. For the two-slope model, the break was set at day 21 for the farms after visual inspection of the raw data. The equation of the two-slope model is shown below:

$$ {\boldsymbol{y}}_{\boldsymbol{i},\boldsymbol{j},\boldsymbol{k}}={\boldsymbol{\theta}}_{\mathbf{0}\boldsymbol{i},\boldsymbol{k}}+{\boldsymbol{\theta}}_{\mathbf{1}\boldsymbol{i},\boldsymbol{k}}\times {\boldsymbol{t}}_{\mathbf{1}\boldsymbol{i},\boldsymbol{j},\boldsymbol{k}}+{\boldsymbol{\theta}}_{\mathbf{2}\boldsymbol{i},\boldsymbol{k}}\times {\boldsymbol{t}}_{\mathbf{2}\boldsymbol{i},\boldsymbol{j},\boldsymbol{k}}+{\boldsymbol{\varepsilon}}_{\boldsymbol{i},\boldsymbol{j},\boldsymbol{k}} $$

where ***y***_***i,j,k***_ is the observed Shannon index or the number of observed OTUs at the ***j***^***th***^ day of calf ***i*** from farm ***k***. ***θ***_**0*i,k***_, ***θ***_**1*i,k***_**,** ***θ***_**2*i,k***_ are the intercept, first slope, and second slope, respectively. ***t***_**1*i,j,k***_ and ***t***_**2*i,j,k***_ represent the time before or on the 21st day and after, respectively, and ***ε***_***i,j,k***_ the residual error.

The farm effect was introduced for each parameter and the calves set as random effects. The LRT was used to compare the fit between the candidate models. For each parameter of the final model, farms were grouped when the effects were not significantly different. We assumed that the random effects and residual errors were independent and had a normal distribution, with a mean of 0. Evaluation of the final model was conducted using basic goodness-of-fit plots.

#### Temporal modelling of the absolute number of *E. coli *per gram of feces

First, we performed Spearman’s correlation between the number of bacteria from the *Escherichia* genus, estimated by quantitative PCR, and the relative abundance of the *Escherichia* genus to assess the consistency of the two techniques. Second, we built linear mixed-effects models to study the temporal dynamics of the absolute number of *E. coli* and tested polynomial functions of time. The farm effect was introduced for each parameter and the calves set as random effects. The equation of the quartic model is shown below:

$$ {\boldsymbol{y}}_{\boldsymbol{i},\boldsymbol{j},\boldsymbol{k}}={\boldsymbol{\theta}}_{\mathbf{0}\boldsymbol{i},\boldsymbol{j},\boldsymbol{k}}+{\boldsymbol{\theta}}_{\mathbf{1}\boldsymbol{i},\boldsymbol{j},\boldsymbol{k}}\times {\boldsymbol{t}}_{\boldsymbol{i},\boldsymbol{j}}+{\boldsymbol{\theta}}_{\mathbf{2}\boldsymbol{i},\boldsymbol{j},\boldsymbol{k}}\times {\boldsymbol{t}}_{\boldsymbol{i},\boldsymbol{j}}^{\mathbf{2}}+{\boldsymbol{\theta}}_{\mathbf{3}\boldsymbol{i},\boldsymbol{j},\boldsymbol{k}}\times {\boldsymbol{t}}_{\boldsymbol{i},\boldsymbol{j}}^{\mathbf{3}}+{\boldsymbol{\theta}}_{\mathbf{4}\boldsymbol{i},\boldsymbol{j},\boldsymbol{k}}\times {\boldsymbol{t}}_{\boldsymbol{i},\boldsymbol{j}}^{\mathbf{4}}+{\boldsymbol{\varepsilon}}_{\boldsymbol{i},\boldsymbol{j}} $$

where ***y***_***i,j,k***_ is the observed number of *E. coli/*g and ***θ***_**0*i,j,k***_, ***θ***_**1*i,j,k***_, ***θ***_**2*i,j,k***_ ***θ***_**3*i,j,k***_, ***θ***_**4*i,j,k***_ the coefficients of each term of the polynomial function of time; ***t***_***i,j***_ the ***j***^***th***^ day of calf ***i***, and ***ε***_***i,j,k***_ the residual error.

The number of random effects was reduced by a backward approach using the Akaike Information Criterion (AIC), starting with the random effect of the coefficient with the highest degree. Selection of the final model and grouping by the farm effect were performed as for the α-diversity indices. We also made the same assumptions of normality and independence and evaluated the final model using basic goodness-of-fit plots.

#### Determination of the influence of antibiotic treatment on temporal predictions of α-diversity indices and the absolute number of *E. coli*

Alterations of fecal microbiota, such as selective depletion of bacterial populations and the reduction of ecological diversity, are generally regarded as gut microbiota dysbiosis markers following antibiotic treatment [63–65]. We investigated such effects on the temporal dynamics of the calves’ fecal microbiota by considering that dysbiosis linked to antibiotic treatment could have occurred for all samples for which a collective antibiotic treatment was given within 15 days before sampling and antibiotic treatment still ongoing or not at the time of sampling (Fig. 1).

The influence of antibiotic treatment on the intercept of α-diversity indices and the absolute number of *E. coli* models was tested by introducing a covariable representing the existence of treatment within the 15 days before sampling. Samples were collected in such a time window on two dates at farm A (days 7 and 21), one date at farm B (day 106), and four dates at farm C (days 7, 21, 35, and 91, Fig. 1).

#### Exploring associations between the abundance of genera and the dose of milk powder

A link between the relative abundance of genera and the dose of milk powder was explored for farms B and C. The Spearman correlation test was used to look for positive associations between the dose of milk powder and the relative abundance of genera. As multiple tests were performed, the *P* values were adjusted using the Bonferroni correction. We searched for the presence of the β-galactosidase enzyme, which hydrolyzes lactose to the monosaccharides glucose and galactose, in the available annotated genomes in the NCBI genome and protein databases for genera that were found to have a significant moderate to strong positive association with the dose of milk powder (r_s_ > 0.4) [37]. We also blasted the protein sequence of the LacZ β-galactosidase enzyme in the NCBI protein database using the program blastp [36, 37].

As *E. coli* is a lactose-fermenting species, we searched for an association between the estimated daily doses of milk powder and the predicted number of *E. coli*/g for farms B and C using Spearman’s correlation test. The farm-predicted numbers of *E. coli*/g were obtained by adding the estimates of the farm parameters to the intercept in our final model.

Means were presented with standard deviations. All statistical analyses were performed using R software (R version 3.1.0) [66]. Mixed effect models were built using nlme [67] and PERMANOVA tests were performed using the “adonis” function in the vegan package [68].

## Supplementary information

**Additional file 1 Fig. S1.** Sequence and OTU distributions after bioinformatics processing. (**a**) Distribution of the number of 16S rRNA gene V4 region sequences in samples after quality filtering. (**b**) Distribution of the number of OTUs in samples after clustering sequences with a similarity cutoff of 97%. The inner lines in the boxplots represent the median, the edges show the first and third quartiles, and the whiskers extend to the 5th and 95th percentiles in (**a**) and (**b**). (**c**) Rarefaction curves for 16S rRNA gene V4 region sequences. Each curve corresponds to a sample. The red vertical line represents the chosen rarefaction threshold.

**Additional file 2 Fig. S2.** Heatmaps of the β-diversity unweighted Unifrac distances matrix for the (**a**) first sampling (day 7), (**b**) second sampling (day 21), and (**c**) last sampling (day 161 for farms A and B and day 147 for farm C). Yellow squares indicate low Unifrac distances, whereas dark red squares indicate high Unifrac distances. Calves are ordered according to farms in both lines and columns. The means ± standard deviations for each sampling on each farm are shown in the lower triangles.

**Additional file 3 Fig. S3.** Observed intra-calf β-diversity weighted Unifrac distances between consecutive samplings for (**a**) farm A, (**b**) farm B, and (**c**) farm C. The dots indicate the Unifrac distances between consecutive samples from the same calf.

**Additional file 4 Fig. S4.** Relative abundance of the five most abundant taxa at the genus level for all calves throughout the fattening period. For each panel, the first and second days represent the sampling date for farm C and farms A and B, respectively. Relative abundance of the five most abundant taxa are given for (**a**) days 35 and 49, (**b**) days 63 and 77, (**c**) days 91 and 106, and (**d**) days 119 and 133. Other detected taxa are depicted by the white bars. Calf IDs are provided at the top of the panels and are ordered according to farms. The color scale of the dots beneath the bar graphs represents the distribution of the Shannon index values. The color key refers to the phylum of each taxa and each palette was built to maximize the distinctiveness between shades.

**Additional file 5 Table S1.** OTUs detected in or absent from previous samples and shared by calves over time. The sampling, ranges of proportions of calves, and OTU taxonomies are presented in each layer. The lists of OTUs consist of (**a**) OTUs simultaneously detected in two consecutive samples in more than 25% of the calves, (**b**) OTUs that were not previously detected and that simultaneously appeared in more than 25% of the calves, and (**c**) OTUs that were simultaneously lost by more than 25% of the calves.

**Additional file 6 Tables S2, S3, and S4.** Tables S2, S3, and S4 contain the estimated parameters for the final models of the Shannon index, the number of observed OTUs, and the absolute number of *E. coli/g,* respectively.

**Additional file 7 Fig. S5.** Dynamics of the mean observed and predicted number of observed OTUs for each farm. Predicted dynamics of the number of observed OTUs, without and with the antibiotic-treatment effect, in the final model are represented in panels (**a**) and (**b**), respectively. The mean values ± standard deviations of the observed data for each farm are represented by the dashed bars. Model-predicted profiles and their 95% confidence bands are represented by the solid lines and bands, respectively. Antibiotic treatments during sampling or within 15 days before sampling are color-coded by farm and indicated above the x-axis in panel (**b**).

**Additional file 8 Table S5.** List of the Spearman’s rank correlation coefficients between genera found in calves’ feces and the dose of milk powder estimated for farms B and C. The genera with a significant positive correlation and a Bonferroni corrected *p-*value < 0.05 are highlighted in green.

**Additional file 9 Fig. S6.** Relative abundance of the genera *Megasphaera*, *Enterococcus, Dialister*, and *Mitsuokella* as a function of the dose of milk powder. Each point represents a sample. These four genera had the highest significant positive correlation with the estimated dose of milk powder in farms B and C. Values on the x-axis correspond to samples in which the corresponding genus was not detected by 16S rRNA gene sequencing.

**Additional file 10 Fig. S7.** Veal calves on fattening farms (**a**) on the first day, corresponding to 14 days of age, and (**b**) at 115 days of age, during the third month of fattening.

**Additional file 11 Table S6**. Absolute number of *Escherichia coli* per gram of feces in all samples, estimated by *Escherichia-*specific quantitative PCR.

## Data Availability

Sequencing reads were deposited as entire raw data in the European Nucleotide Archive repository (ENA) under the BioProject ID PRJEB33072, separately for each sample. The code used for processing the 16S rRNA gene sequences was originally developed by Kozich et al. [57] and is available at the website https://www.mothur.org/wiki/MiSeq_SOP (access on January 2017). The unrarefied OTU table and the corresponding taxonomic classification analyzed during the current study are available from the corresponding author upon reasonable request. The *Escherichia-*specific qPCR dataset has been included as Additional file 11: Table S6.

## Abbreviations

AIC: Akaike Information Criterion
ANSES: French Agency for Food, Environmental and Occupational Health & Safety
ESBL: Extended spectrum β-lactamase
LRT: Likelihood ratio test
OTU: Operational taxonomic unit
PERMANOVA: Permutational multivariate analysis of variance
qPCR: Quantitative PCR
rRNA: Ribosomal ribonucleic acid
