# Supplementary figures and images for: Temporal dynamics of the fecal microbiota in veal calves in a 6-month field trial
Source: Anim Microbiome. 2020 Sep 15;2:32. doi: 10.1186/s42523-020-00052-6 (PMC7807794; doi:10.1186/s42523-020-00052-6)

## Slide 1
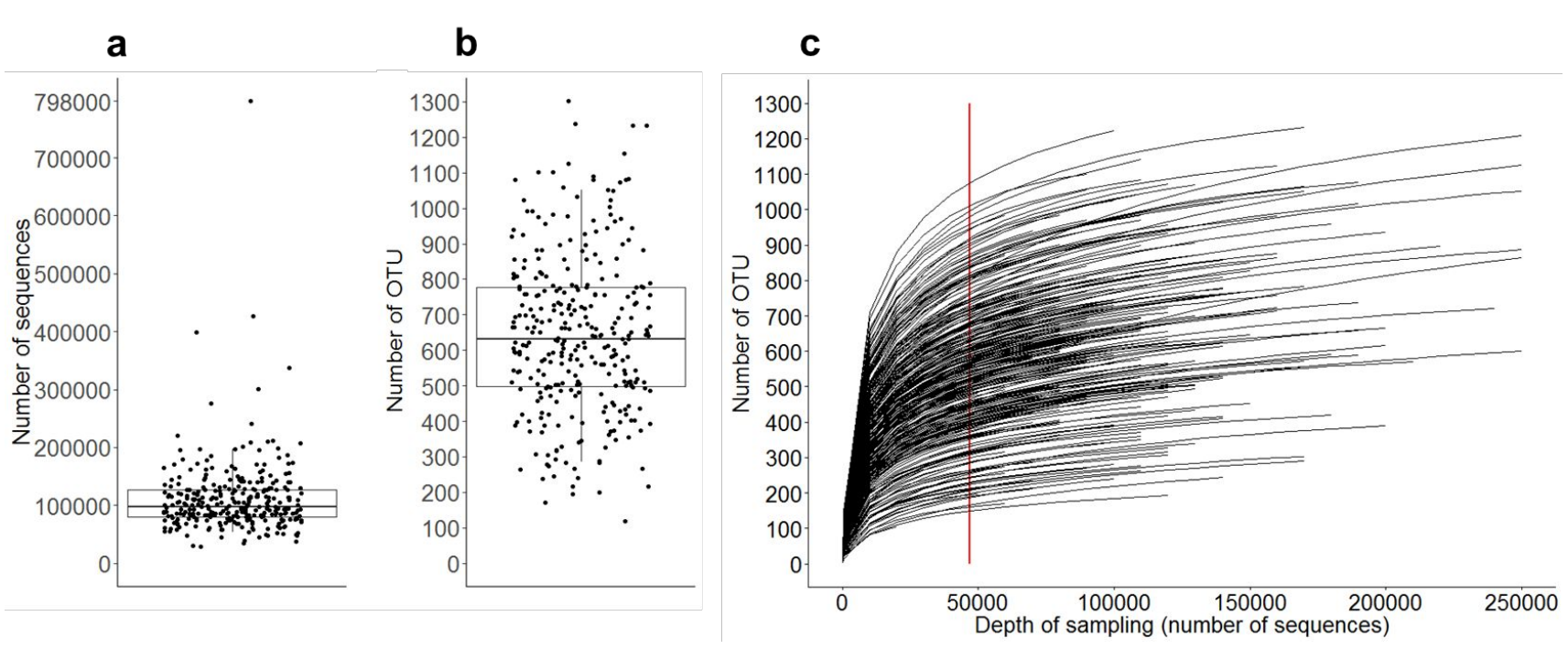

Supplement: Supplementary file 1 — Additional file 1 Fig. S1. Sequence and OTU distributions after bioinformatics processing. (a) Distribution of the number of 16S rRNA gene V4 region sequences in samples after quality filtering. (b) Distribution of the number of OTUs in samples after clustering sequences with a similarity cutoff of 97%. The inner lines in the boxplots represent the median, the edges show the first and third quartiles, and the whiskers extend to the 5th and 95th percentiles in (a) and (b). (c) Rarefaction curves for 16S rRNA gene V4 region sequences. Each curve corresponds to a sample. The red vertical line represents the chosen rarefaction threshold. [file 42523_2020_52_MOESM1_ESM.pptx]

## Slide 1
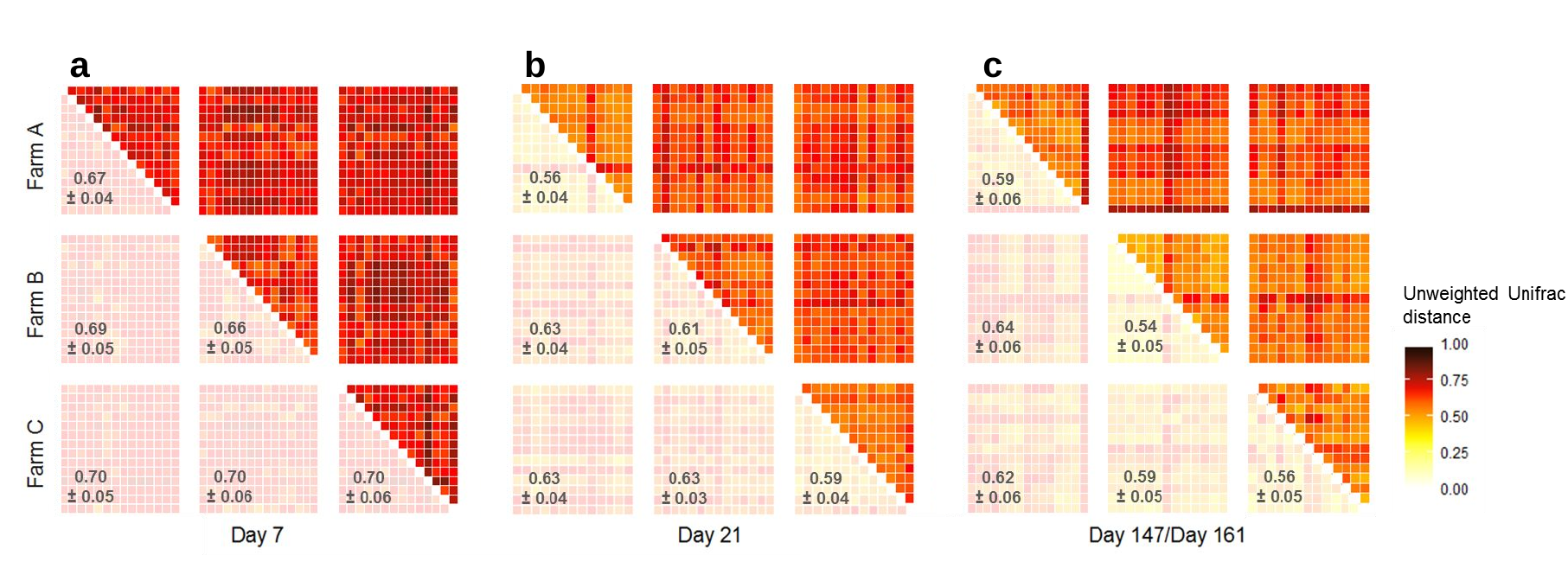

a
b
c

Supplement: Supplementary file 2 — Additional file 2 Fig. S2. Heatmaps of the β-diversity unweighted Unifrac distances matrix for the (a) first sampling (day 7), (b) second sampling (day 21), and (c) last sampling (day 161 for farms A and B and day 147 for farm C). Yellow squares indicate low Unifrac distances, whereas dark red squares indicate high Unifrac distances. Calves are ordered according to farms in both lines and columns. The means ± standard deviations for each sampling on each farm are shown in the lower triangles. [file 42523_2020_52_MOESM2_ESM.pptx]

## Slide 1
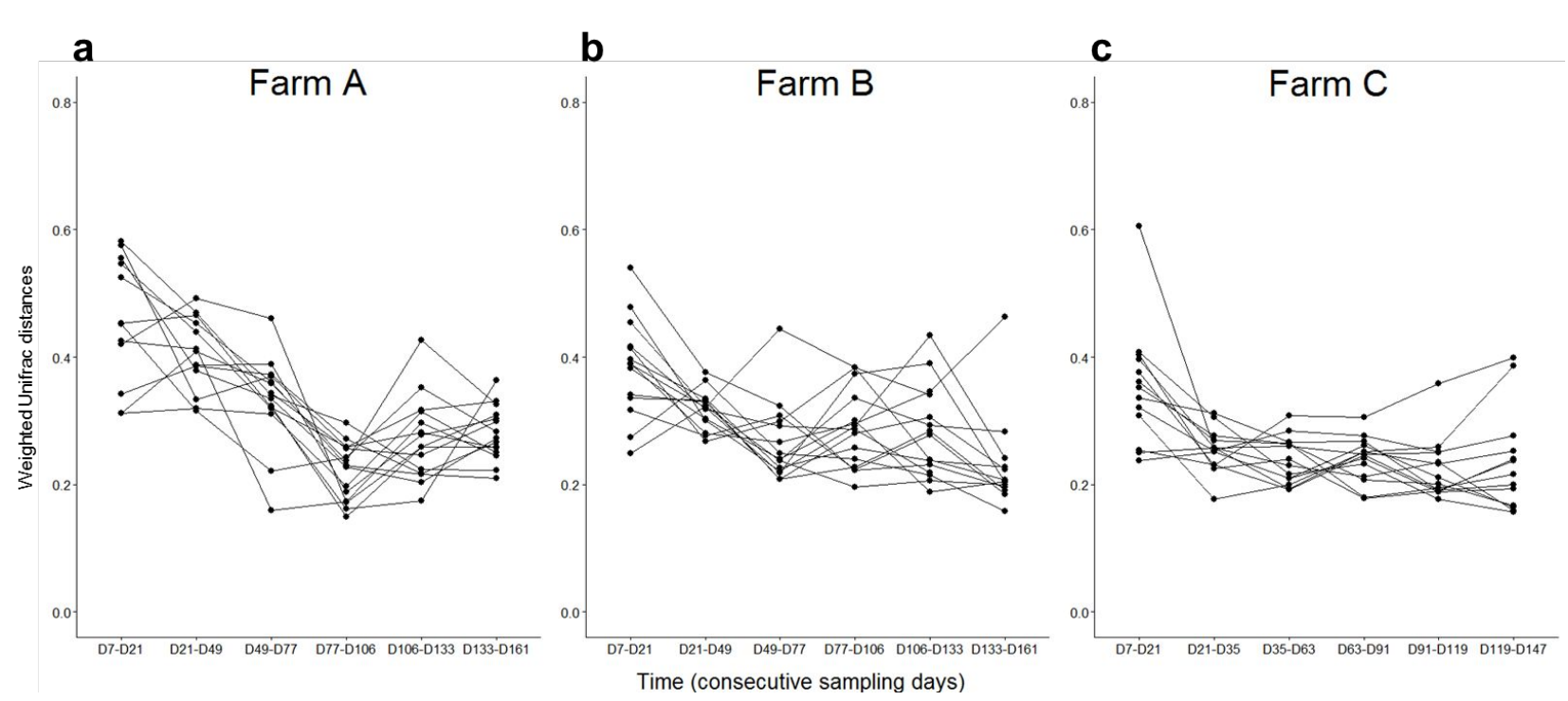

Supplement: Supplementary file 3 — Additional file 3 Fig. S3. Observed intra-calf β-diversity weighted Unifrac distances between consecutive samplings for (a) farm A, (b) farm B, and (c) farm C. The dots indicate the Unifrac distances between consecutive samples from the same calf. [file 42523_2020_52_MOESM3_ESM.pptx]

## Slide 1
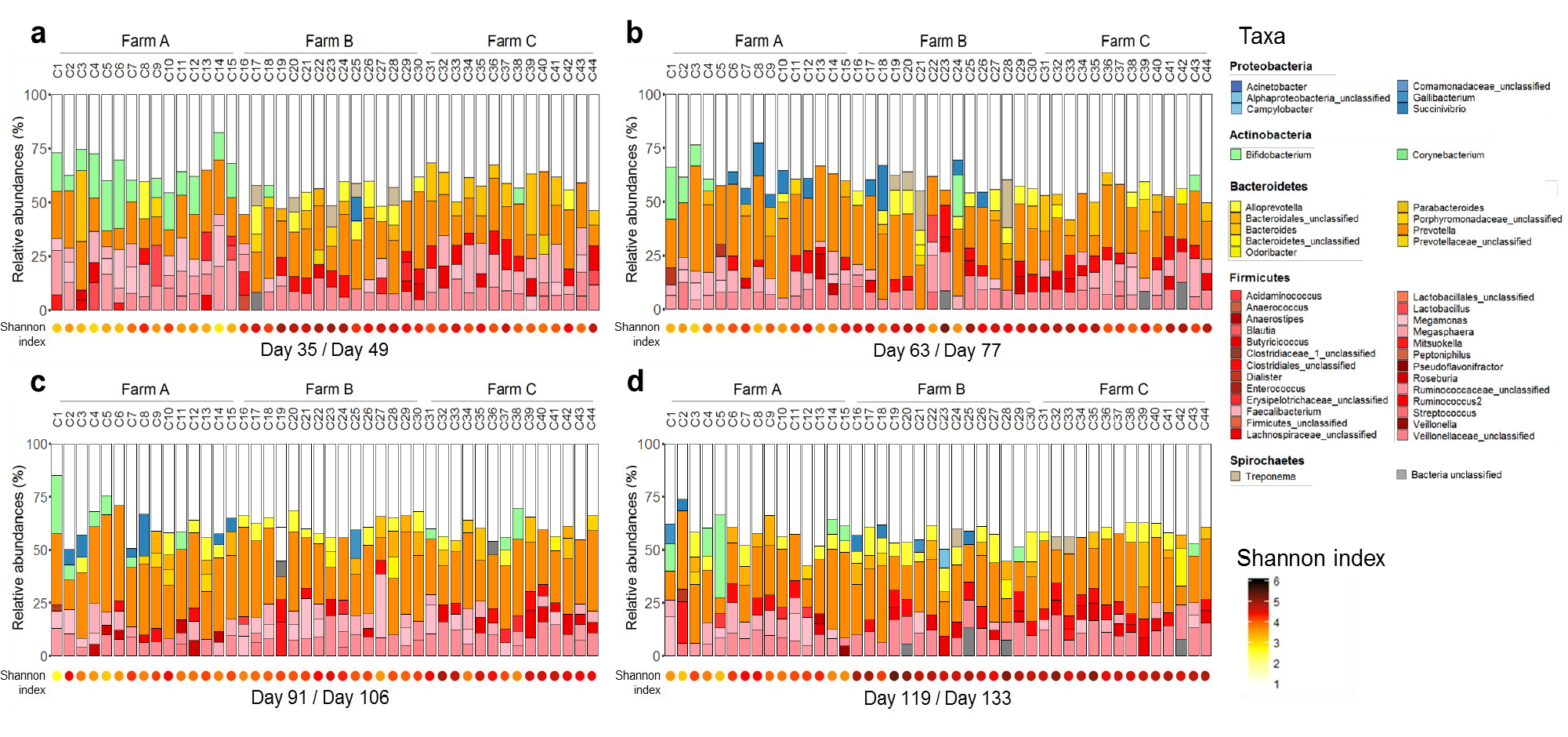

Supplement: Supplementary file 4 — Additional file 4 Fig. S4. Relative abundance of the five most abundant taxa at the genus level for all calves throughout the fattening period. For each panel, the first and second days represent the sampling date for farm C and farms A and B, respectively. Relative abundance of the five most abundant taxa are given for (a) days 35 and 49, (b) days 63 and 77, (c) days 91 and 106, and (d) days 119 and 133. Other detected taxa are depicted by the white bars. Calf IDs are provided at the top of the panels and are ordered according to farms. The color scale of the dots beneath the bar graphs represents the distribution of the Shannon index values. The color key refers to the phylum of each taxa and each palette was built to maximize the distinctiveness between shades. [file 42523_2020_52_MOESM4_ESM.pptx]

## Slide 1
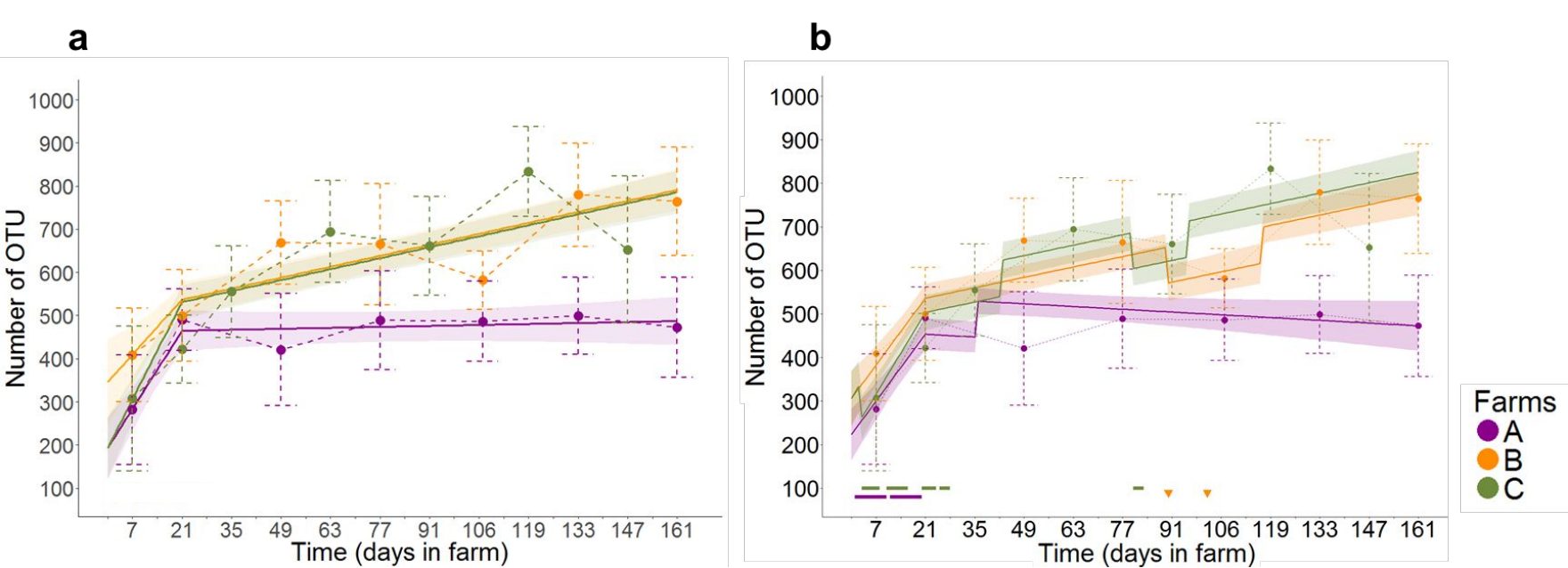

Supplement: Supplementary file 7 — Additional file 7 Fig. S5. Dynamics of the mean observed and predicted number of observed OTUs for each farm. Predicted dynamics of the number of observed OTUs, without and with the antibiotic-treatment effect, in the final model are represented in panels (a) and (b), respectively. The mean values ± standard deviations of the observed data for each farm are represented by the dashed bars. Model-predicted profiles and their 95% confidence bands are represented by the solid lines and bands, respectively. Antibiotic treatments during sampling or within 15 days before sampling are color-coded by farm and indicated above the x-axis in panel (b). [file 42523_2020_52_MOESM7_ESM.pptx]

## Slide 1
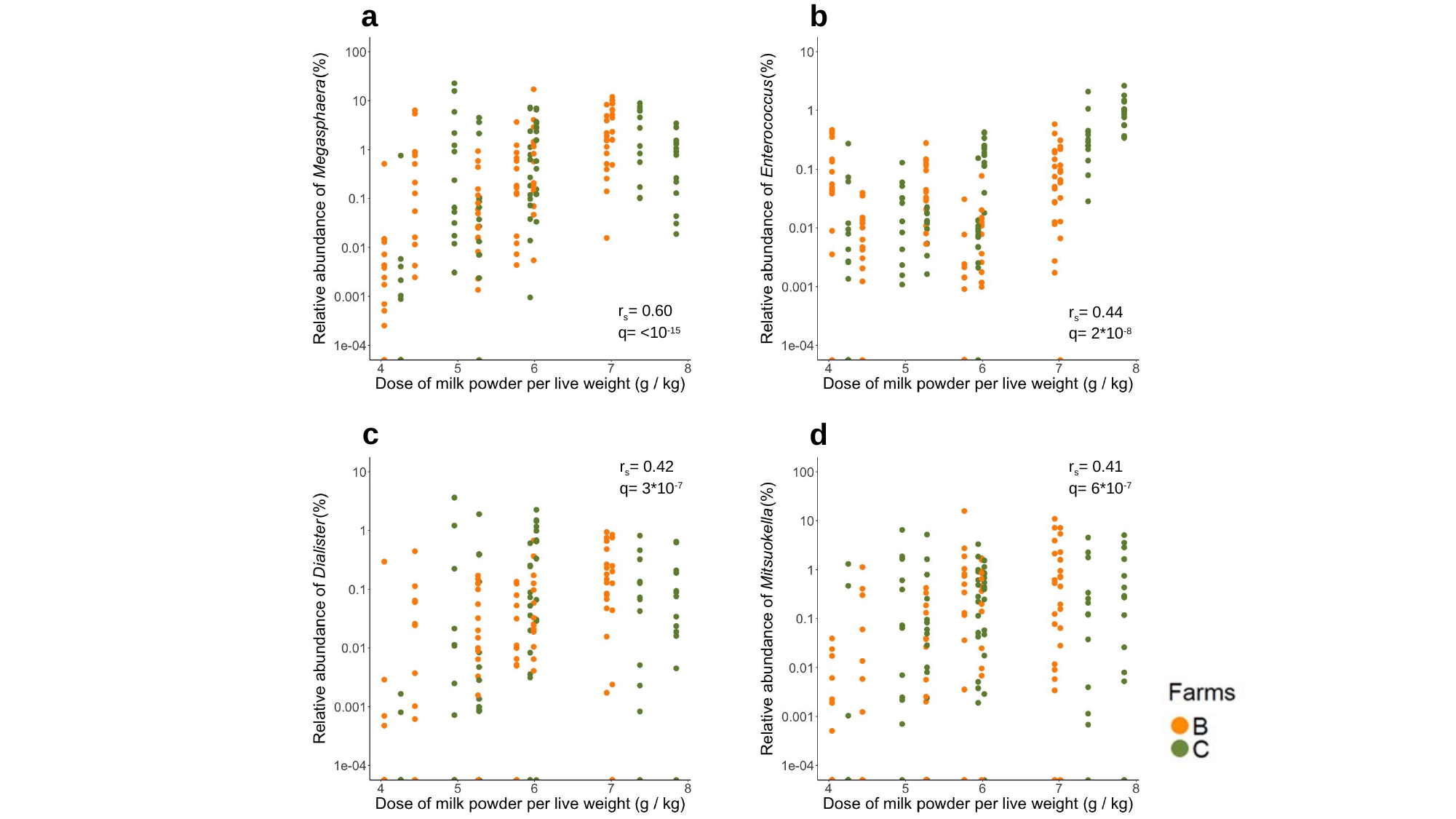

a
b
rs= 0.60
q= <10-15
rs= 0.44
q= 2*10-8
c
d
rs= 0.42
q= 3*10-7
rs= 0.41
q= 6*10-7

Supplement: Supplementary file 9 — Additional file 9 Fig. S6. Relative abundance of the genera Megasphaera, Enterococcus, Dialister, and Mitsuokella as a function of the dose of milk powder. Each point represents a sample. These four genera had the highest significant positive correlation with the estimated dose of milk powder in farms B and C. Values on the x-axis correspond to samples in which the corresponding genus was not detected by 16S rRNA gene sequencing. [file 42523_2020_52_MOESM9_ESM.pptx]

## Slide 1
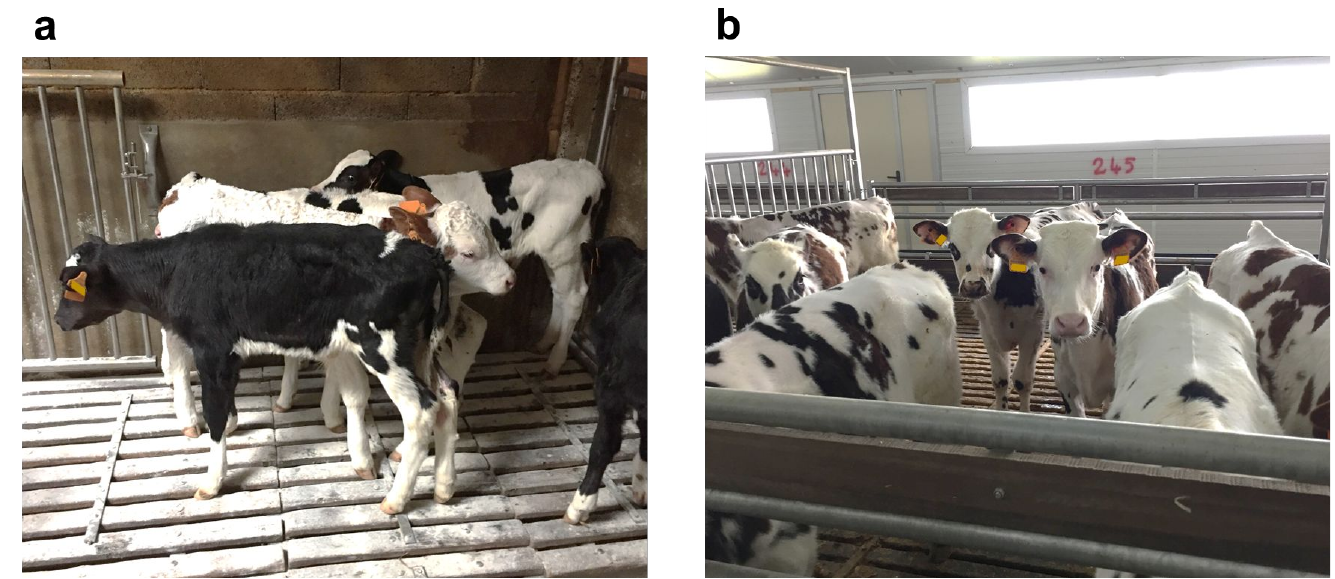

Supplement: Supplementary file 10 — Additional file 10 Fig. S7. Veal calves on fattening farms (a) on the first day, corresponding to 14 days of age, and (b) at 115 days of age, during the third month of fattening. [file 42523_2020_52_MOESM10_ESM.pptx]
