# Supplementary material for: Temporal dynamics of the fecal microbiota in veal calves in a 6-month field trial
Source: Anim Microbiome. 2020 Sep 15;2:32. doi: 10.1186/s42523-020-00052-6 (PMC7807794; doi:10.1186/s42523-020-00052-6)
Supplement: Supplementary file 6 — Additional file 6 Tables S2, S3, and S4. Tables S2, S3, and S4 contain the estimated parameters for the final models of the Shannon index, the number of observed OTUs, and the absolute number of E. coli/g, respectively. [file 42523_2020_52_MOESM6_ESM.docx]

Table S2. Estimated parameters for the Shannon index final model, with and without antibiotic treatment.

| Parameters/coefficients | Final model without antibiotic treatment | | |  | Final model with antibiotic treatments | | |
| --- | --- | --- | --- | --- | --- | --- | --- |
|  | Estimates | CI_95%_ | p value |  | Estimates | CI_95%_ | p value |
| **Intercept** |  |  |  |  |  |  |  |
| **μ_0_ (farm A)** | 2.07 | [1.68; 2.46] | **< 10^-15^** |  | 2.44 | [2.08; 2.79] | **< 10^-15^** |
| **β_0.B_** | 1.14 | [0.49; 1.80] | **0.0008** |  | - | - | - |
| **β_0.C_** | 0.35 | [-1.44; -0.13] | **0.003** |  | - | - | - |
| **β_0.BC_** | - | - | - |  | 0.40 | [0.21; 0.58] | **6×10^-5^** |
| **β_0.antibiotic_** | - | - | - |  | -0.17 | [-0.27; -0.06] | **0.003** |
| **ω_0_** | 0.85 | [0.63; 1.16] | - |  | 0.89 | [0.56; 1.17] | - |
| **First slope** |  |  |  |  |  |  |  |
| **μ_1_ (farm B)** | 0.044 | [0.019; 0.070] | **0.0007** |  | 0.07 | [0.05; 0.08] | **2×10^-10^** |
| **β_1.AC_** | 0.035 | [0.004; 0.067] | **0.03** |  | - | - | - |
| **ω_1_** | 0.038 | [0.026; 0.055] | - |  | 0.04 | [0.025; 0.054] | - |
| **Second slope** |  |  |  |  |  |  |  |
| **μ_2_ (farm A)** | 0.0006 | [-0.0011; 0.0023] | 0.5 |  | -8.9×10^-5^ | [-0.0012; 0.0017] | 0.9 |
| **β_2.BC_** | 0.0032 | [0.0010; 0.0053] | **0.004** |  | 0.003 | [0.0011; 0.0056] | **0.003** |
| **ω_2_** | 0.0009 | [0.0001; 0.0108] | - |  | 0.001 | [0.0002; 0.0264] | - |
| **σ** | 0.37 | [0.33; 0.41] | - |  | 0.36 | [0.32; 0.40] | - |

CI_95%_: 95% confidence interval, μ: estimated parameter. The farm reference for each parameter is shown in brackets. β: farm and antibiotic effects on related parameter, ω: random effect standard deviation, σ: residual standard deviation.

Table S3. Estimated parameters for the number of observed OTUs final model, with and without antibiotic treatment.

| Parameters/coefficients | Final model without antibiotic treatment | | |  | Final model with antibiotic treatments | | |
| --- | --- | --- | --- | --- | --- | --- | --- |
|  | Estimates | CI_95%_ | p value |  | Estimates | CI_95%_ | p value |
| **Intercept** |  |  |  |  |  |  |  |
| **μ_0_ (farm B)** | 345.4 | [246.6; 444.1] | **< 10^-15^** |  | 306.3 | [242.5; 369.6] | **2×10^-14^** |
| **β_0.AC_** | -152.4 | [-274.3; -30.4] | **0.02** |  | - | - | - |
| **β_0.antibiotic_** | - | - | - |  | -82.7 | [-115.8; -49.6] | **1×10^-6^** |
| **ω_0_** | 92.7 | [0.63; 1.16] | - |  | 101.3 | [39.9; 170.4] | - |
| **First slope** |  |  |  |  |  |  |  |
| **μ_1_ (farm A)** | 12.95 | [8.84; 17.07] | **< 10^-15^** |  | 11.0 | [7.8; 14.1] | **3×10^-10^** |
| **β_1.B_** | -3.83 | [-10.25; 2.57] | 0.2 |  | - | - | - |
| **β_1.C_** | 3.15 | [0.22; 6.08] | **0.04** |  | 2.4 | [0.14; 4.74] | **0.04** |
| **ω_1_** | 2.73 | [0.55; 13.56] | - |  | 2.9 | [0.8; 7.4] | - |
| **Second slope** |  |  |  |  |  |  |  |
| **μ_2_ (farm A)** | 0.16 | [-0.37; 0.69] | 0.6 |  | -0.45 | [-0.96; 0.04] | 0.07 |
| **β_2.BC_** | 1.66 | [1.00; 2.32] | **< 10^-15^** |  | 2.2 | [1.6; 2.7] | **9×10^-10^** |
| **ω_2_** | 0.40 | [0.10; 1.67] | - |  | 0.46 | [0.09; 0.85] | - |
| **σ** | 111.3 | [101.2; 122.3] | - |  | 107.0 | [94.0; 114.0] | - |

CI_95%_: 95% confidence interval, μ: estimated parameter. The farm reference for each parameter is shown in brackets. β: farm and antibiotic effects on related parameter, ω: random effect standard deviation, σ: residual standard deviation.

Table S4. Estimated parameters for the final model of the absolute number of *E. coli,* with and without antibiotic treatments.

| Parameters/coefficients | Final model without antibiotic treatment | | |  | Final model with antibiotic treatments | | |
| --- | --- | --- | --- | --- | --- | --- | --- |
|  | Estimates | CI_95%_ | p value |  | Estimates | CI_95%_ | p value |
| **Constant term** |  |  |  |  |  |  |  |
| **μ_0_** | 6.57 | [6.15; 6.99] | **< 10^-15^** |  | 7.07 | [6.49; 7.65] | **< 10^-15^** |
| **β_0.antibiotic_** | - | - | - |  | -0.37 | [-0.66; -0.08] | **0.01** |
| **Linear term** |  |  |  |  |  |  |  |
| **μ_1_ (farms A&C)** | 0.18 | [0.14; 0.22] | **< 10^-15^** |  | 0.17 | [1.29; 2.13] | **3×10^-14^** |
| **β_1.B_** | -0.07 | [-0.11; -0.03] | **0.0003** |  | -0.08 | [-0.12; -0.04] | **9×10^-5^** |
| **Quadratic term** |  |  |  |  |  |  |  |
| **μ_2_ (farms A&C)** | -0.0042 | [-0.005; -0.003] | **2×10^-13^** |  | -0.0041 | [-0.0052; 0.0031] | **4×10^-13^** |
| **β_2.B_** | 0.0024 | [0.001; 0.004] | **0.0003** |  | 0.0025 | [0.0012; 0.0037] | **0.0002** |
| **Cubic term** |  |  |  |  |  |  |  |
| **μ_3_ (farms A&C)** | 3.27×10^-5^ | [2.26×10^-5^; 4.28×10^-5^] | **7×10^-10^** |  | 3.26 ×10^-5^ | [2.26×10^-5^; 4.25 ×10^-5^] | **6×10^-10^** |
| **β_3.B_** | -2.29×10^-5^ | [-3.61×10^-6^; -9.78 ×10^-6^] | **0.0007** |  | -2.32×10^-5^ | [-3.61×10^-5^; -1.02×10^-5^] | **0.0006** |
| **Quartic term** |  |  |  |  |  |  |  |
| **μ_4_ (farms A&C)** | -8.27×10^-08^ | [-1.13×10^-7^; -5.20×10^-8^] | **2×10^-7^** |  | -8.31×10^-8^ | [-1.14×10^-7^; -5.27×10^-8^] | **2×10^-7^** |
| **β_4.B_** | 6.82×10^-08^ | [2.63×10^-8^; 1.10×10^-7^] | **0.002** |  | 6.73×10^-8^ | [2.58×10^-8^; 1.08×10^-7^] | **0.002** |
| **σ** | 0.83 |  | - |  | 0.82 | - | - |

CI_95%_: 95% confidence interval, μ: estimated parameter. The farm reference for each parameter is shown in brackets. β: farm and antibiotic effects on related parameter, ω: random effect standard deviation, σ: residual standard deviation. The unit is the log_10_ (number of *E. coli* / g of feces).
